# A highly mutable *GST* is essential for bract colouration in *Euphorbia pulcherrima* Willd. Ex Klotsch

**DOI:** 10.1186/s12864-021-07527-z

**Published:** 2021-03-23

**Authors:** Vinicius Vilperte, Robert Boehm, Thomas Debener

**Affiliations:** 1 Institute of Plant Genetics, Leibniz Universität Hannover, 30419 Hannover, Germany; 2 Present address: KWS SAAT SE & Co. KGaA, 37574 Einbeck, Germany; 3 Klemm + Sohn GmbH & Co., 70379 Stuttgart, KG Germany

**Keywords:** Anthocyanin, *Euphorbia pulcherrima*, Ionizing radiation, Glutathione S-transferase, Mutation breeding, Poinsettia, Short repeat sequences

## Abstract

**Background:**

Mutation breeding is an extraordinary tool in plant breeding to increase the genetic variability, where mutations in anthocyanin biosynthesis are targets to generate distinctive phenotypes in ornamental species. In poinsettia, ionizing radiation is routinely applied in breeding programs to obtaining a range of colours, with nearly all pink and white varieties being obtained after γ- or X-ray mutagenesis of red varieties. In the present study we performed a thorough characterization of a potential mutagenesis target gene as the main responsible for the ‘*white paradox*’ in poinsettia.

**Results:**

We identified a *GST* gene in poinsettia (*Bract1*) as an essential factor for the expression of anthocyanin-based red colouration of bracts, which presents a high phylogenetic similarity to known anthocyanin-related GSTs. Red poinsettia varieties and white mutants generated from these varieties by X-ray were analysed for polymorphisms related to the ‘*white paradox*’ in the species. A 4 bp mutation in a short repeat within the coding region of *Bract1* is most likely responsible for the appearance of white phenotypes upon irradiation treatment. The polymorphism between wild-type and mutant alleles co-segregates with the phenotype in progeny from heterozygous red and white parents. Moreover, overexpression of *Bract1* wild-type allele in Arabidopsis *tt19* mutants restored the anthocyanin phenotype, while the *Bract1* mutated allele showed to be non-functional.

**Conclusions:**

The identified repeat seems to be highly unstable, since mutated plants can be easily detected among fewer than 200 shoots derived from 10 mutated plants. Our data indicate that particular short repeat sequences, similar to microsatellite sequences or so-called dynamic mutations, might be hot spots for genetic variability. Moreover, the identification of the *Bract1* mutation fills a gap on the understanding on the molecular mechanism of colour formation in poinsettia.

**Supplementary Information:**

The online version contains supplementary material available at 10.1186/s12864-021-07527-z.

## Background

Poinsettia, *Euphorbia pulcherrima* Willd. ex Klotsch, commonly known as Christmas Star, is an important ornamental crop, especially due to its association with Christmas time in North America, Europe, and Asia, with annual sales reaching nearly 150 million dollars in the USA [69]. Its ornamental value is based on its intensely coloured bracts, which can be red, white, pink, or yellow or even have dual, scattered, or marbled colourations. Nonetheless, poinsettia breeding still focuses on red- and white-coloured varieties due to higher acceptance by consumers. In 2018, in Germany, approximately 80% of the poinsettias grown were red, 11% were white, and 9% were pink or had dual/scattered colouration [70].

Ionizing radiation is an important tool in mutation breeding for new colour variations in poinsettia, with nearly all pink and white varieties being obtained after gamma or X-ray mutagenesis of shoots of red varieties. Poinsettia mutation breeding is usually performed on cuttings that are irradiated with moderate dosages (~ 20 Gy), and mutants are selected on side shoots of the originally irradiated shoots. Flowering induction in the species occurs under short-day conditions and is accompanied by the development and colouration of bracts. Therefore, green leaves and red bracts occur concomitantly and accumulate different groups of pigments, i.e., chlorophylls and anthocyanins [53, 61]. Several anthocyanin types have been identified in poinsettia bracts and are responsible for its colouration range [3, 55, 66]; however, molecular information is still limited for the species [28, 72]. Nonetheless, genes responsible for the biosynthesis of the anthocyanin pathway have been intensively characterized in a range of species, with its regulation being highly dependent on R2R3-MYB regulatory genes and MYB-bHLH-WD40 (MBW) regulatory complexes [16, 58, 76].

Once synthesized on the cytoplasmic surface of the endoplasmic reticulum (ER), anthocyanin molecules need to be stored in the vacuole to prevent oxidation and loss of colour [4]. Two main models of anthocyanin transport have been proposed: i) a vesicle trafficking-mediated model, where vesicle-like structures filled with anthocyanins are imported into the central vacuole via vesicle fusion [23, 27, 62]; and ii) a transporter-mediated model, where anthocyanins are carried across the vacuolar membrane by transport proteins (e.g., ABC and MATE transporters) with the help of glutathione *S*-transferase (GST) enzymes [26, 63, 78]. GSTs can bind to anthocyanin molecules to form a complex, thus escorting them from the ER to the vacuole, preventing oxidation [13, 54, 67, 78]. Anthocyanin-related GSTs play major roles in anthocyanin transport, since loss of function of these proteins leads to phenotypes with a lack of pigmentation, such as *bz2* (*Bronze-2*) in maize, *an9* (*Anthocyanin 9*) in petunia, *tt19* (*Transparent Testa 19*) in Arabidopsis, *fl3* (*Flavonoid3*) in carnation, *riant* (*regulator involved in anthocyanin transport*) in peach, and *rap* (*reduced anthocyanin in petioles*) in strawberry [2, 8, 38, 43, 48]. In our previous study, an anthocyanin-related GST-like gene showed higher expression in a red poinsettia variety than in the white counterpart, thus making it a promising candidate responsible for the so-called *‘white paradox’*, e.g. appearance of acyanic (uncolored) phenotype despite the detection of expression of all structural genes and the related enzyme activities involved in the formation of red anthocyanin pigments [72].

In our current study, we identified an anthocyanin-related *GST* as the most likely target of the radiation-induced mutation of red poinsettias in white bract sports. Using different approaches, this study demonstrates the functionality of the poinsettia *GST* as an anthocyanin transporter. Most importantly, we show that a short repeat motif within the coding region of the gene is highly unstable upon mutation treatment, which leads to the high frequency of anthocyanin mutations observed in commercial mutation breeding. In addition to facilitating mutation breeding for bract colours, these results may be a starting point for analysing the genetic instability of short repeat sequences in plants.

## Results

### Identification and characterization of *Bract1*

**Fig. 1:**
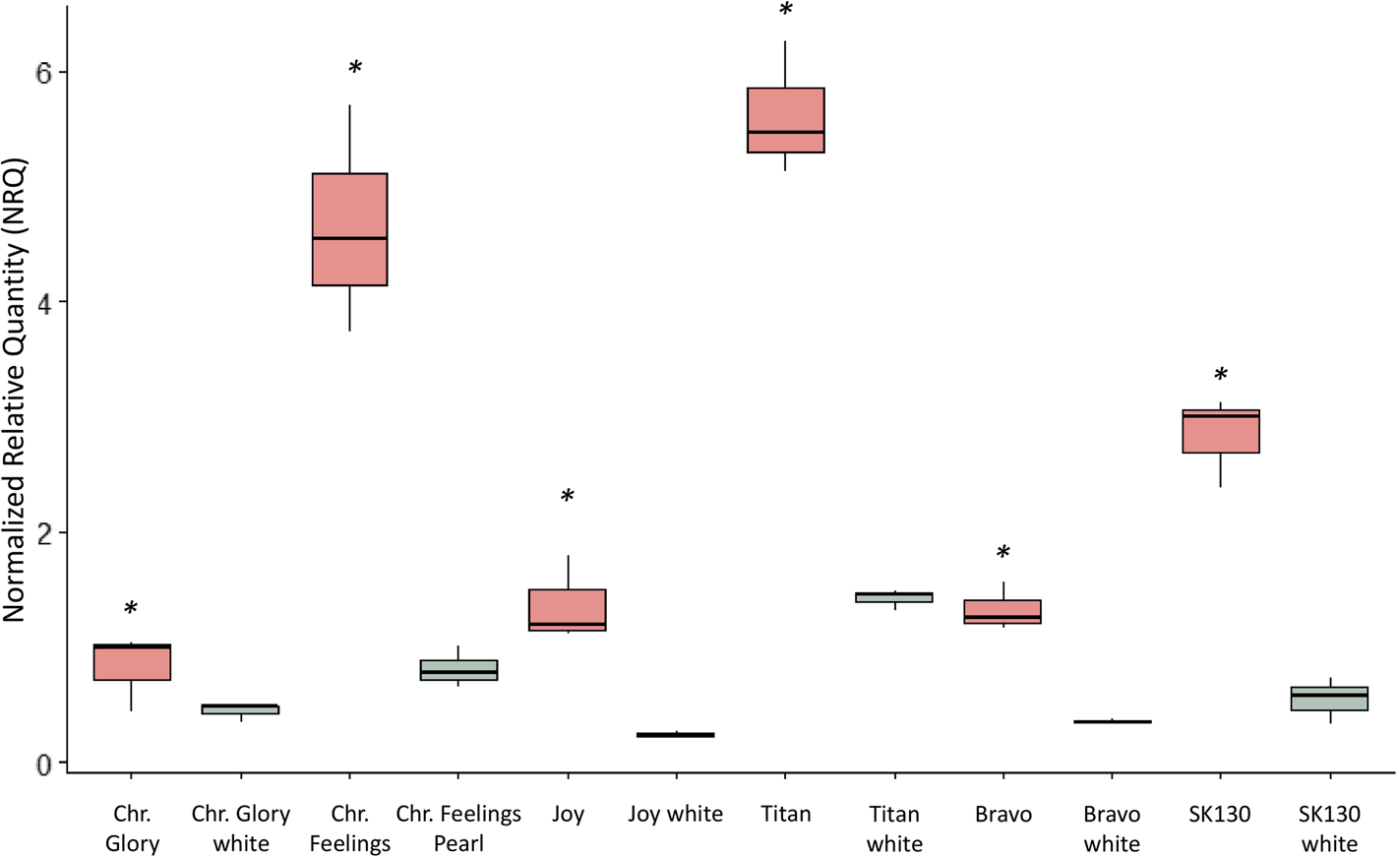
RT-qPCR of *Bract1* for six pairs of red-bracted poinsettia varieties and their independently generated white mutants. The normalized relative quantity (NRQ) was calculated according to the Pfaffl equations [59] and using the ‘Chr. Glory’ variety as a reference sample. The ‘*’ symbol indicates significant differences calculated with REST software between red and white pairs at *p* ≤ 0.05

In a previous study [72], we observed higher expression of an anthocyanin-related *GST-like* gene (termed *Bract1* hereafter) in the red poinsettia variety ‘Christmas Feelings’ than in its white counterpart ‘Christmas Feelings Pearl’. To investigate whether a similar phenomenon is observed in other red and white poinsettia pairs, we performed RT-qPCRs for six pairs of red-bracted poinsettia varieties and their independently generated white mutants. Normalized relative quantity (NRQ) values were calculated relative to one of the biological replicates of the ‘Chr. Glory’ variety according to the Pffafl method and equations [59]. The levels of *Bract1* expression varied among all varieties, with the varieties ‘Christmas Feelings’, ‘Titan’ and ‘SK130’ showing the highest relative expression. Although no lack of expression was observed in any of the white varieties, all red varieties showed significantly higher expression of *Bract1* than their white counterparts (Fig. 1).

**Fig. 2:**
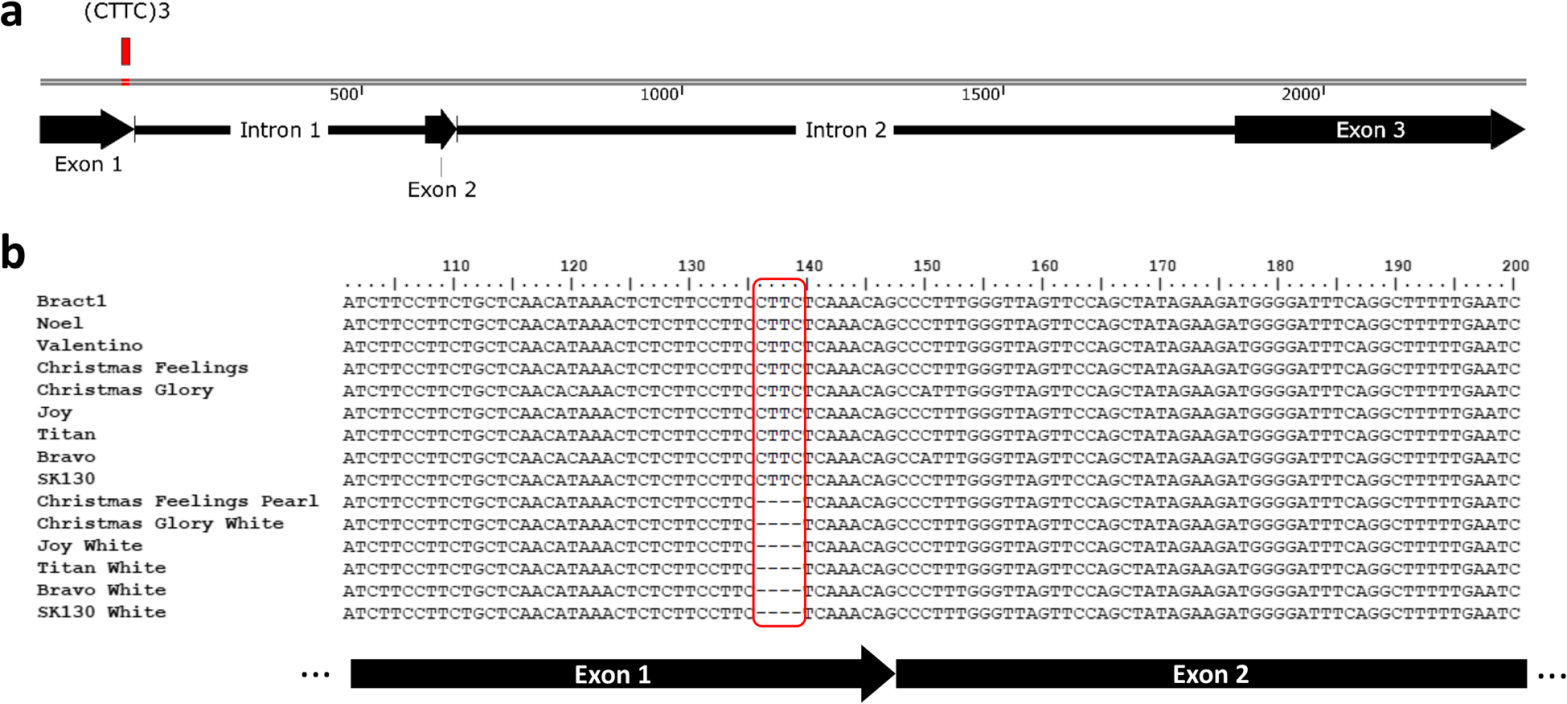
Characterization of the anthocyanin-related GST gene (*Bract1*) in *Euphorbia pulcherrima*. **a** Schematic representation of the full-length sequence (2314 bp) of *Bract1* in the ‘Vintage’ variety. Black arrows represent the exonic regions. Black lines represent the intronic regions. The red square represents the location of the tetranucleotide motif SSR locus (CTTC_3_). **b** Nucleotide alignment of the *Bract1* CDS for 14 red- and white-bracted poinsettia varieties. The figure shows a 100 bp region of the CDS in which a 4 bp deletion (red box) is observed only in the white varieties. Black arrows below the sequences show the location of the sequences in each exon. The first sequence corresponds to *Bract1* from the ‘Vintage’ variety and was used as a reference for the alignment

To further characterize the anthocyanin-related *GST* in poinsettia we sequenced the complete coding and intronic regions of the gene for the ‘Vintage’ variety. The final full-length (from first ATG start codon to stop codon) *Bract1* sequence contains 2314 bp, with three exons (147 bp, 48 bp and 450 bp) and two introns (455 bp and 1214 bp) (Fig. 2a). The coding sequences (CDSs) of *Bract1* from 14 red- and white-bracted poinsettia varieties (‘Noel’, ‘Valentino’, ‘Christmas Feelings’, ‘Christmas Feelings Pearl’, ‘Christmas Glory’, ‘Christmas Glory White’, ‘Joy’, ‘Joy White’, ‘Titan’, ‘Titan White’, ‘Bravo’, ‘Bravo White’, ‘SK130’ and ‘SK130 White’) were further sequenced. The sequence alignment showed high similarity in the CDS for all varieties, except for six single-nucleotide polymorphisms (SNPs) that were identified in both the ‘Chr. Glory’ and ‘Bravo’ varieties (Table 1). This result shows the presence of at least two allelic forms of the *Bract1* gene. Additionally, a 4 bp deletion located 8 bp upstream of the first exon-intron junction was observed in all white varieties (Fig. 2b). The deletion is located in a short repeat locus, resembling a short simple sequence repeat (SSR), with a tetranucleotide motif ((CTTC)_3_) composition. The exact location of the (CTTC)_3_ motif is shown in Fig. 1a. The full-length gene sequence and CDS are available in Additional File S1.

**Table 1:** List of SNPs identified in the sequenced varieties in comparison to the Vintage variety

Position	Original	Alternative	Varieties
90	T	A	Chr. Glory/Bravo
120	T	C	Chr. Glory/Bravo
150	C	A	Chr. Glory/Bravo
525	C	T	Chr. Glory/Bravo
578	A	G	Chr. Glory/Bravo
604	C	A	Chr. Glory/Bravo

*Bract1* encodes a putative functional protein of 215 amino acids (aa) and a mass of 24.6 kDa, with distinctive GST components: a conserved GSH-binding site (G-site) located in the N-terminal domain and a C-terminal substrate-binding domain (H-site) [14]. The predicted protein from the CDS containing the 4 bp deletion is a putative truncated protein with an early stop codon at position aa52 due to a frameshift in the mRNA. The full-length amino acid sequence and the truncated version are available in Additional File S1.

### Bract colouration associated with a deletion in the *Bract1* gene

The colour range in poinsettia varieties is obtained either through classic breeding (crossing) or mutagenic breeding (radiation), thus generating a spectrum of bract colours, such as pink, marble, orange and white/creamy. The white varieties are often obtained through radiation mutagenesis of the red varieties, followed by shoot development and trait selection. Therefore, red and white poinsettias from the same variety are referred to as ‘pairs’ due to their highly similar genetic background. However, not all red varieties can produce white sports through radiation. Therefore, red poinsettia varieties are distinguished into ‘heterozygous’ and ‘homozygous’ for the colouration locus according to their ability to generate white sports and according to the segregation of red and white phenotypes in progeny of crosses with white genotypes.

**Fig. 3:**
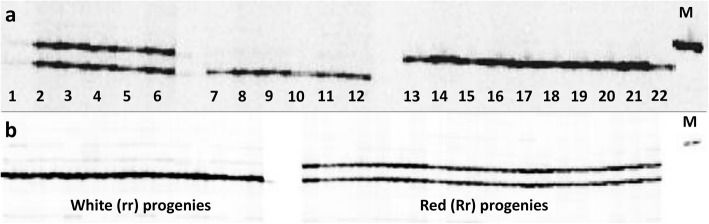
PCR amplification of the tetranucleotide motif SSR locus (CTTC)_3_ from the *Bract1* gene. **a** Band patterns from the amplified PCR fragments for *Bract1* in 22 red- and white-bracted poinsettia varieties. Samples 1–6 correspond to red heterozygous varieties, samples 7–12 correspond to white varieties, and samples 13–22 correspond to red homozygous varieties. **b** Example of the amplified PCR fragments for *Bract1* for the segregating population ‘Joy’ (Rr) x ‘Joy White’ (rr). M = marker. Figures were cropped for better visualization. Full length figures are available in Additional File S2

Since the 4 bp *indel* in the SSR locus of *Bract1* had shown indications of polymorphism among the different poinsettia varieties—and a correlation with bract colouration—we used a genotyping approach based on the fluorescent labelling of PCR fragments. We genotyped 22 different poinsettia varieties bearing red and white bracts (Fig. 3a, Additional File S2). All the red heterozygous varieties showed two distinct copies of the allele (with and without the 4 bp deletion), while their white counterparts showed only the copy with the deletion. On the other hand, homozygous red varieties (i.e., those unable to generate white sports) showed only the copy without the deletion.

We further genotyped a segregating population with 190 progeny from ‘Joy’ (Rr) x ‘Joy White’ (rr) containing 36 white and 154 red plants (Fig. 3b, Additional File S2). Contrary to expectation, we observed a deviation in the segregation ratio, which was approximately 4:1 (red:white), instead of the expected 1:1 ratio for this crossing. This may be explained by the fact that seeds from white varieties are less vital than those from red varieties (von Tubeuf, Selecta One, pers. comm.). In addition, white varieties also exhibit lower pollen fertility, thus increasing the chances of self-pollination when red varieties are used as a female parent (von Tubeuf, Selecta One, pers. comm.). In fact, 17 red progeny showed only the wild-type copy of the allele (data not shown), which can be attributed only to self-pollination. Nonetheless, all the white progeny showed only the allele copy containing the deletion, thus reinforcing our hypothesis that the presence of the allele containing the deletion in a homozygous recessive state is correlated with the white phenotype.

### *Bract1* is the anthocyanin-related GST orthologue in poinsettia

**Fig. 4:**
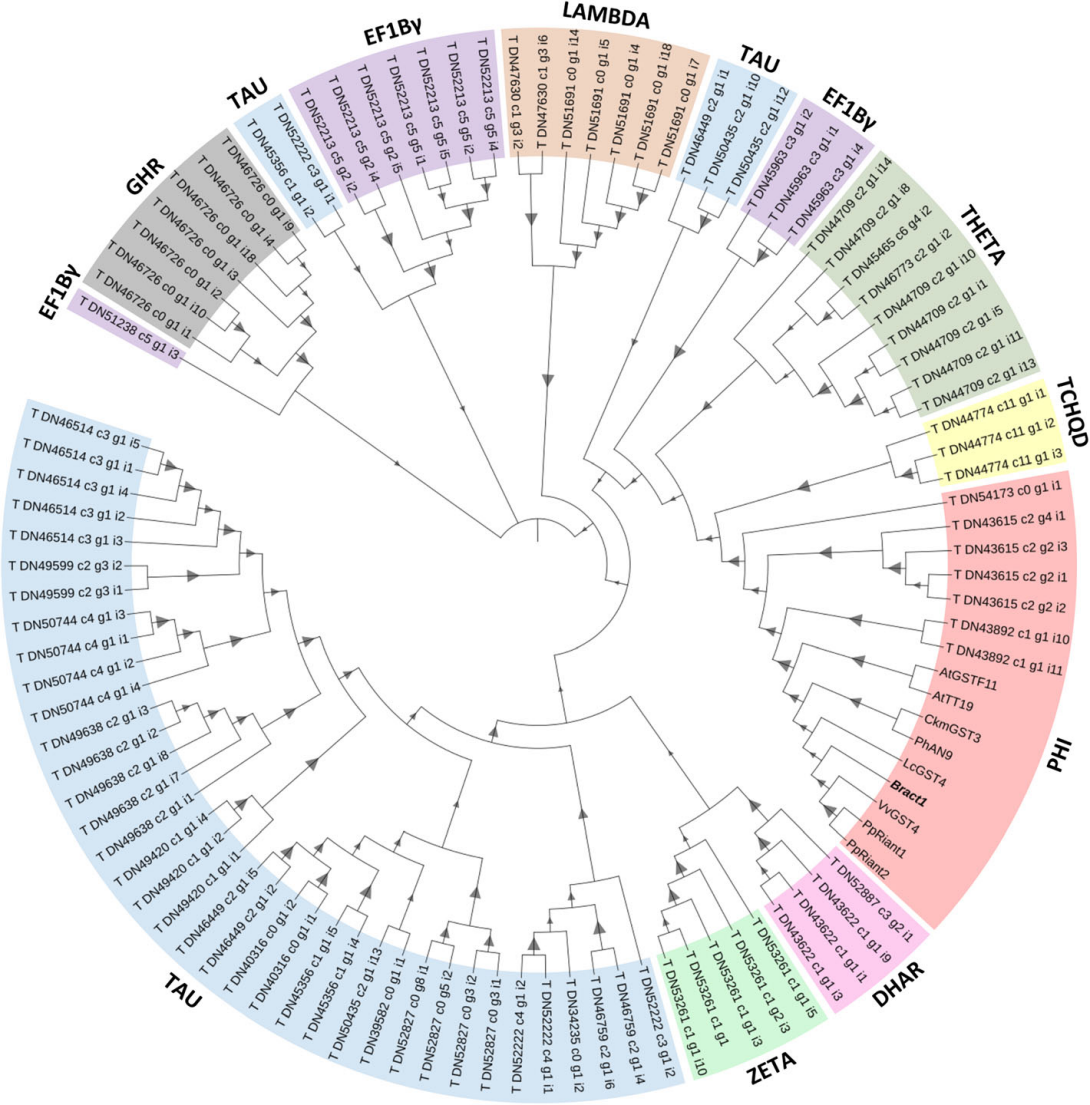
Phylogenetic tree for 96 poinsettia GSTs and anthocyanin-related GSTs from other plant species. Amino acid sequences were aligned using MUSCLE. The maximum likelihood (ML) method based on the WAG matrix-based model was used to calculate the phylogenetic tree. Phylogenetic testing was performed using the bootstrap method with 1000 replicates, which are depicted as triangles, where the smallest value represents 1.3% and the largest 100%. Branch lengths were omitted for better visualization

As GST genes occur in large gene families, we wanted to analyse whether the poinsettia GST gene was related to other GST genes involved in anthocyanin transport to the vacuole. Therefore, we computed a phylogenetic tree from the deducted amino acid sequences of 95 GST family members from our previously assembled poinsettia transcriptome [72], as well as the *Bract1* and anthocyanin-related GSTs from other species (*CkmGST3*, *LcGST4*, *VvGST4*, *PhAN9*, *PpRiant1*, *PpRiant2*, *AtGSTF11* and *AtTT19*). Nine GST classes were identified among the poinsettia GSTs: Tau, Theta, Lambda, Zeta, Phi, tetrachlorohydroquinone dehalogenase (TCHQD), glutathionyl hydroquinone reductase (GHR), dehydroascorbate reductase (DHAR) and eukaryotic translation elongation factor 1B-γ (Ef1Bγ). Except Tau and Ef1Bγ, all other GST classes showed a single cluster (Fig. 4). All anthocyanin-related GSTs belong to the Phi class and clustered together in the phylogenetic tree, with *Bract1* showing high similarity with these GSTs.

**Fig. 5:**
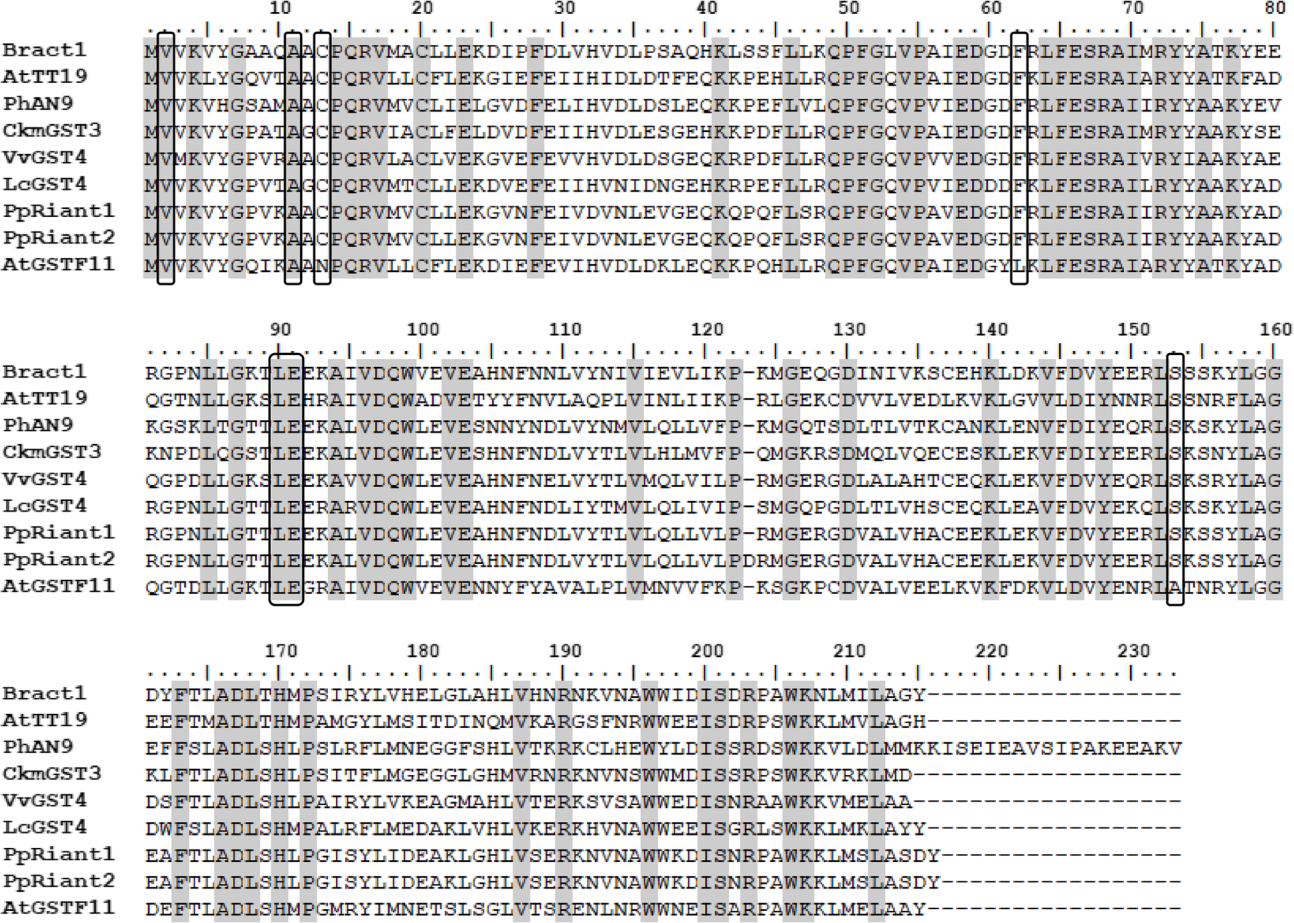
Protein sequence alignment of BRACT1 and anthocyanin-related GSTs from other plant species. The numbers in the alignments indicate the amino acid positions, and black boxes show amino acids that are known to be conserved in anthocyanin-related GSTs [32, 37, 40]. Sequences were aligned using the ClustalW function in the BioEdit Sequence Alignment Editor v7.2.5

By aligning the *Bract1* nucleotide CDSs with those of anthocyanin-related GSTs from other species, an overall nucleotide similarity of 61.9% was observed (Additional File S3). Protein alignment of BRACT1 with the other anthocyanin-related GSTs resulted in an overall similarity of 58.3%, with the peach RIANT1 protein showing the highest similarity (66.5%) (Additional File S3). Interestingly, we identified seven amino acid residues, previously reported as specific to anthocyanin-related GSTs [32, 37, 40], that are conserved in the protein alignment, except in AtGSTF11: 2Val, 11Ala, 13Cys, 62Phe, 90Leu, 91Glu and 152Ser (Fig. 5). In summary, these results indicate that *Bract1* is the anthocyanin-related GST orthologue in poinsettia.

### *Bract1* functionally complements the Arabidopsis *tt19* mutant phenotype

**Fig. 6:**
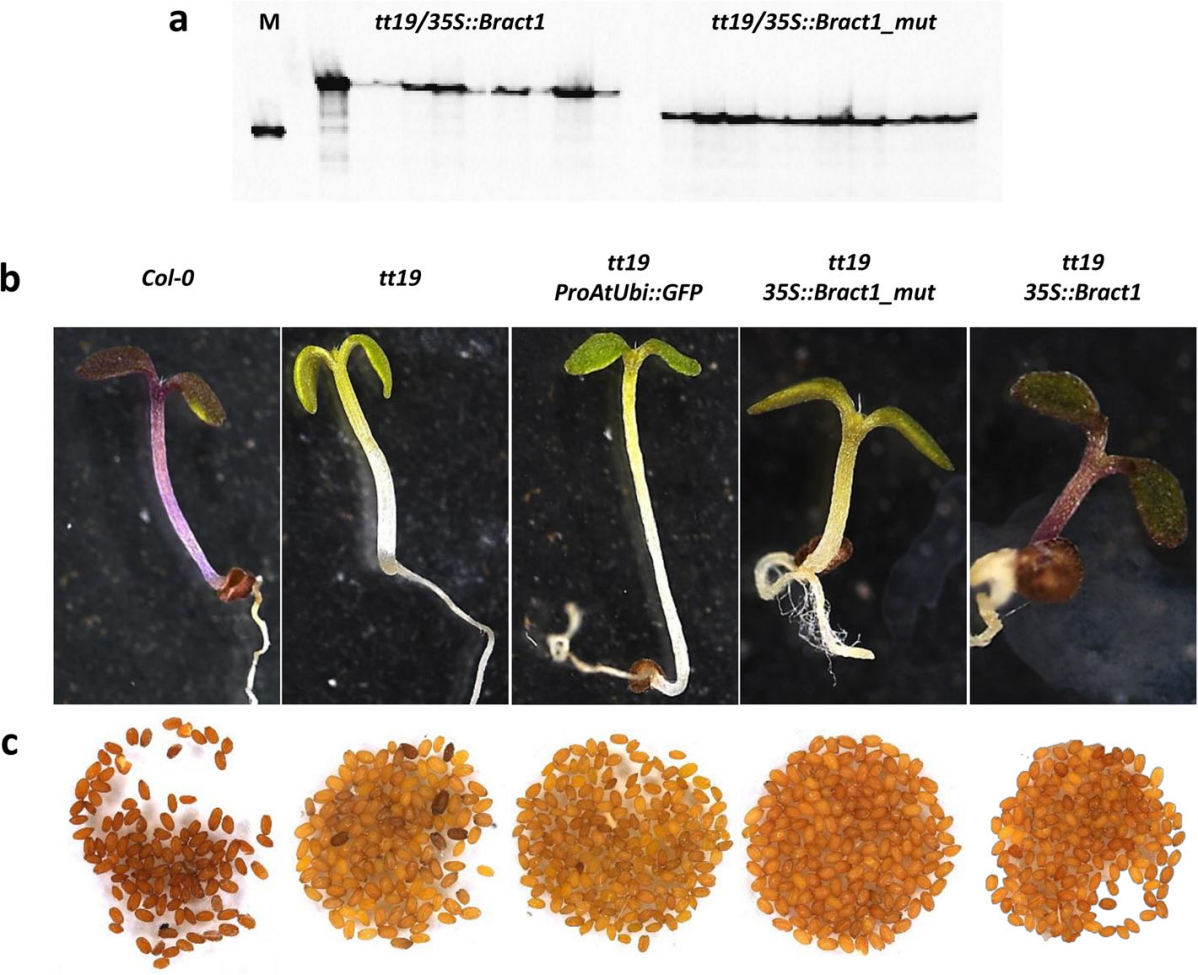
Functional complementation of the Arabidopsis *tt19* mutant with the *Bract1* gene. **a** Genotyping of 10 independent biological replicates from the T_2_ progeny of *tt19/35S::Bract1* and *tt19/35S::Bract1_mut* transgenic plants. Figure was cropped for better visualization. Full length figure is available in Additional File S2. **b** Phenotypes of seedlings (14 days old) and C) mature seeds of *Col-0* and *tt19* and the transgenic lines *tt19*/*ProAtUbi::GFP*, *tt19*/*35S::Bract1* and *tt19*/*35S::Bract1_mut* in the tt19 background

To examine the in vivo function of *Bract1* as an anthocyanin transporter, we tested the ability of *Bract1* cDNA to functionally complement the Arabidopsis GST mutant *tt19,* which is defective in the expression of anthocyanins in aboveground organs and seeds. Two constructs containing the *Bract1* cDNA (with and without the 4 bp deletion) under the cauliflower mosaic virus (CaMV) 35S promoter were introduced into the *tt19* mutant by the floral-dip method [11, 75]. Although the constructs contained a GFP marker for the selection of transgenic events, we genotyped 10 independent biological replicates from the T_2_ progeny of *tt19/35S::Bract1* and *tt19/35S::Bract1_mut* transgenic plants. All progeny contained the correct allele from the *Bract1* gene, thus confirming the correct integration of the transgenic construct (Fig. 6a, Additional File S2).

Upon stimulation of anthocyanin accumulation in seedlings by irradiation with red/blue LEDs, the *tt19/35S::Bract1* transgenic lines displayed a purple hypocotyl phenotype at the seedling stage, similar to the Columbia (*Col-0*) line but not the *tt19* mutant (Fig. 6b). On the other hand, *tt19/35S::Bract1* transgenic lines did not show complementation of the anthocyanin phenotype. The *ProAtUbi::GFP* construct, used as a control for infiltration, did not result in any phenotypic changes. Moreover, transgenic plants harbouring *Bract1* did not complement the seed colour of *tt19*, as the seed colour at the ripening stage remained the same as that of the mutant *tt19* in transgenic plants (Fig. 6c). This finding suggests that *Bract1* may have distinct functions from *TT19* during seed coat pigmentation. Taken together, these results not only emphasize the role of *Bract1* in anthocyanin transport in poinsettia but also demonstrate that a deletion in its coding region leads to a colourless phenotype.

### De novo mutations occur with high frequency and include deletion of the 4 bp repeat

To study the stability of the 4 bp repeat within the first exon of the *Bract1* gene, we analysed DNA samples from mutation experiments conducted over the last 4 years at Selecta One. In brief, 10 cuttings from the varieties ‘Aurora’, ‘SK159 Dark Pink’, ‘Aurora Jingle’ and ‘SK183’ were X-ray irradiated with 20 Gy (30 Gy for ‘SK183), and subsequently, side shoots from those cuttings were further propagated. DNA was extracted and analysed as previously described from 377, 191, 188 and 186 of the propagated side shoots. Table 2 shows the results indicating that out of 942 samples, 9 mutations could be detected. Three mutated progeny were identified in both ‘SK159 Dark Pink’ and ‘Aurora Jingle’ individuals and two in the ‘SK183’ individuals, and only one mutated individual was identified in the ‘Aurora’ progeny. Unfortunately, as this was part of a commercial breeding programme, individual shoots were not labelled in a way that would allow tracing them back to one of the original shoots that were irradiated. However, even if all the mutations detected in each of the separate mutation treatments were redundant and originated from one original mutational event, the frequency was extraordinarily high.

**Table 2:** Fragment analysis of progeny from three X-ray-irradiated poinsettia varieties. Two methods were used for the fragment analysis: polyacrylamide gel electrophoresis (PAGE) and fragment length analysis (FLA) by capillary electrophoresis

Variety/year of irradiation	Number of progeny				Type of analysis
	Total	Homozygous (RR)	Heterozygous (Rr)	Homozygous (rr)	
Aurora/2016	377	376	1	0	PAGE
SK159 Dark Pink/2018	191	187	3	0	FLA
Aurora Jingle/2018	188	185	3	0	FLA
SK183/2018	186	1	184	1	FLA

### Microsatellite repeats are not an anthocyanin-related feature

**Fig. 7:**
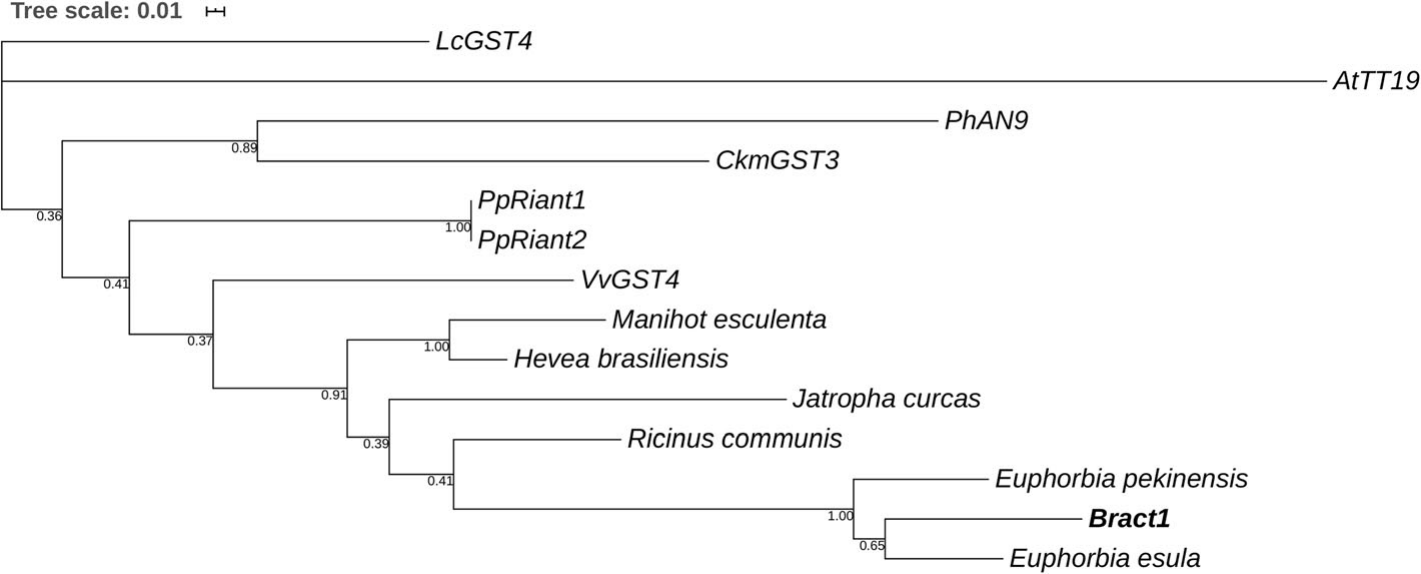
Phylogenetic tree of anthocyanin-related GSTs and GST-like genes from Euphorbiaceae species. CDS nucleotide sequences were aligned using MUSCLE. The maximum likelihood (ML) method based on the T92 matrix-based model was used to calculate the phylogenetic tree. Phylogeny testing was performed using the bootstrap method with 1000 replicates, which are depicted next to the branches

The microsatellite repeat present in the *Bract1* gene shows signs of instability upon irradiation treatment. To identify whether such repeats are a common feature for anthocyanin-related GSTs in Euphorbiaceae or related taxa or are a family-specific feature, we first computed a phylogenetic tree from the CDSs of *Bract1*, known anthocyanin-related GSTs (*CkmGST3*, *LcGST4*, *VvGST4*, *PhAN9*, *PpRiant1*, *PpRiant2*, and *AtTT19*) and GST-like orthologues from Euphorbiaceae species (*Euphorbia esula*, *Euphorbia pekinensis*, *Ricinus communis*, *Jatropha curcas*, *Hevea brasiliensis* and *Manihot esculenta*). Figure 7 shows that *Bract1* shared high similarity with the GSTs from the two *Euphorbia* species (*E. esula* and *E. pekinensis)* but also closely clustered with the GSTs from the other Euphorbiaceae species. Although none of the GST-like genes from other Euphorbiaceae have been investigated as putative anthocyanin transporters, they may perform a similar function due to their homology with *Bract1* and the other anthocyanin-related GSTs.

**Fig. 8:**
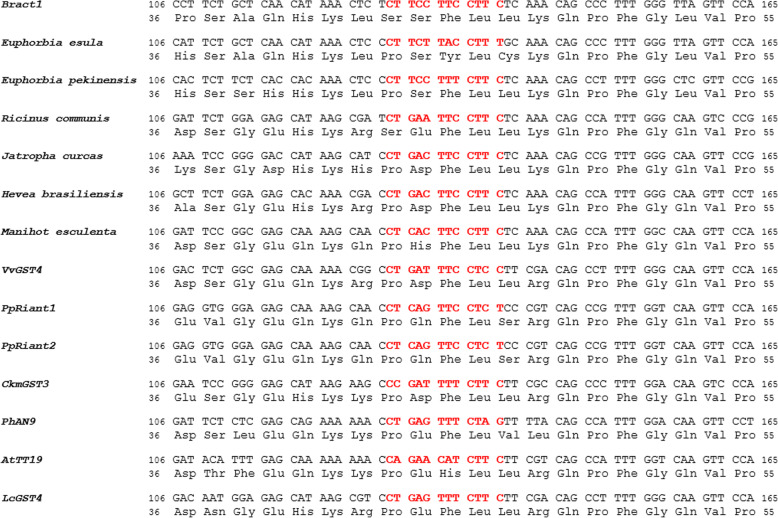
Partial sequence alignment of *Bract1*, anthocyanin-related GSTs and orthologue GSTs from Euphorbiaceae species. The alignment spans a 60 bp region of the CDS containing the (CTTC)_3_ SSR motif (represented in red)**.** The numbers in the alignments indicate the nucleotide and amino acid positions in the CDS. Sequences were aligned using the ClustalW function in the BioEdit Sequence Alignment Editor v7.2.5. The complete alignment is available in Additional File S7

Furthermore, we wanted to assess the distribution of the microsatellite repeat and possibly investigate its origin. The sequence alignment shows that none of the GSTs analysed contain the same (CTTC)_3_ SSR motif observed in *Bract1* (Fig. 8). When analysing the GSTs from Euphorbiaceae species, two CTTC repeat units were present in *E. pekinensis*, with a single nucleotide substitution responsible for the loss of the third repeat. *J. curcas*, *H. brasiliensis* and *M. esculenta* showed two CTTC repeat units, with two nucleotide substitutions related to the loss of the third repeat. Last, *E. esula* and *R. communis* showed a single CTTC unit and three nucleotide substitutions in the microsatellite region. The anthocyanin-related GSTs showed greater distinction in the microsatellite region. Three of the genes contained a single CTTC repeat unit (*CkmGST3*, *AtTT19* and *LcGST4*), while the others did not contain any repeat unit (*VvGST4*, *PpRiant1*, *PpRiant2* and *PhAN9*), which was due to the higher number of nucleotide substitutions (ranging from 4 to 7 substitutions). In conclusion, even though some of the known anthocyanin-related GSTs contain a CTTC sequence, CTTC repeats seem to be a common feature of Euphorbiaceae GSTs, and the three repeats from the *E. pulcherrima* GST (*Bract1*) behave in an unstable manner upon being subjected to ionizing irradiation.

## Discussion

Anthocyanins, a class of flavonoid secondary metabolite compounds [47], are responsible for providing orange to blue colours in plant tissues, and their biosynthetic and regulatory mechanisms have been widely characterized [76]. However, there is still debate on the mechanism of anthocyanin transport from the ER to the vacuole [62, 63]. Strong evidence for the involvement of transport proteins [21, 24], with a special role of GST enzymes, has been reported in several plant species (Alfenito et al. [38, 43];). In the present study, we demonstrated that the *Bract1* gene functions as an anthocyanin transporter in poinsettia and that a highly mutable repeat in its coding region leads to frequent deletions and therefore to a colour-deficient phenotype.

### *Bract1* is a functional GST gene related to anthocyanin transport in poinsettia

GSTs are a large and diverse group of enzymes with multifunctional roles, especially in the detoxification of xenobiotics as well as in responses to biotic and abiotic stresses [1, 15]. The classification of GSTs is based on sequence conservation, genomic organization, and physiochemical properties, among other features [18, 33, 42]. Based on our previous study [72], we identified 95 *GST* genes in poinsettia and phylogenetically classified them into nine different classes based on their similarity with known Arabidopsis GSTs (Fig. 4). To date, 14 GST classes have been identified in plants: tau (U), phi (F), lambda (L), DHAR, theta (T), zeta (Z), EF1Bγ, TCHQD, microsomal prostaglandin E-synthase type 2 (mPGES-2), GHR, metaxin, Ure2p, hemerythrin (H) and iota (I) (reviewed by [42]).

A large number of GSTs have been identified in plant species, such as 49 in *Capsella rubella* *[*31*]*, 55 in Arabidopsis (), 61 in Citrus [45] and 139 in *L. chinensis* *[*32*]*. *Bract1* clusters with high bootstrap support with anthocyanin-related GSTs from other species (e.g., *AtTT19*, *PhAN9* and *VvGST4*), with all of these GSTs belonging to the phi class. Known anthocyanin-related GSTs belong almost exclusively to the phi class, except for *Bronze-2* from maize, which belongs to the tau class [50]. Further support for *Bract1* being a member of the phi-type plant GST genes is provided by the presence of two introns as a characteristic of this group of genes, such as *AN9* from petunia and *TT19* from Arabidopsis (Alfenito et al. [54];).

Complementation studies using Arabidopsis *tt19* mutants have been widely applied as proof of concept for the function of GSTs as anthocyanin transporters (Alfenito et al. [32, 34, 37, 40, 46, 54, 57];). Due to the high amino acid conservation of GST enzymes involved in flavonoid accumulation among species [77], they can complement each other’s anthocyanin-deficient mutants (Alfenito et al. [43];). However, similar to our observation for *Bract1*, not all of these genes complemented both the shoot and seed phenotypes [34, 40, 48]. TT19 is involved in both anthocyanin accumulation in vegetative tissues and proanthocyanidin (PA) accumulation in Arabidopsis seed coats, which provides its brown colouration [38]. Transgenic *tt19* mutants overexpressing the petunia AN9 orthologue showed *AN9* mRNA expression in developing siliques, but the seed colour still remained the same as the wild-type mutant [38]. Altogether, these results suggest that GST orthologues from different species may have distinct functions from TT19 during seed-coat pigmentation.

A direct complementation of poinsettia white mutants with the functional *Bract1* would ultimately prove its function in bract colouration. However, neither *Agrobacterium*-mediated infiltration nor biolistic particle delivery system (a.k.a. gene gun) were successful for transient expression studies (data not shown). Stable transformation in poinsettia have been done using electrophoresis-based methods [9, 71], but no stable transgenic poinsettia was obtained. Successful stable transformation via *Agrobacterium*-mediated infiltration has been previously achieved, but the process is time-consuming [10]. Attempts to perform stable transformation of poinsettia with *Bract1* alleles will bridge the current knowledge gap but are out of the scope of the present study.

### A loss-of-function mutation in *Bract1* is the cause of the “*white paradox*” in poinsettia

Based on our results, we hypothesize that deletion of one unit of the repeat in the *Bract1* gene is responsible for most of the white genotypes in poinsettia. This hypothesis is strongly supported by the evidence that the *tt19/35S::Bract1_mut* mutant was not able to complement the anthocyanin phenotype in the Arabidopsis *tt19* mutant, unlike the *tt19/35S::Bract1* mutant. Mutations in GSTs leading to colourless phenotypes have been previously reported. A mutation in the *fl3* gene in carnation leads to a light pink phenotype, but a brighter phenotype is observed upon complementation by petunia *AN9* and maize *Bz2* *[*43*]*. In peach, four alleles of a GST gene (*Riant*) were identified, with two of them containing frameshift mutations and unable to complement the Arabidopsis *tt19* phenotype. Varieties containing copies of the mutated alleles in a homozygous state showed flowers with white variegated phenotypes [8]. Last, a single-nucleotide polymorphism (SNP) in the strawberry *RAP* gene, leading to a premature stop codon, results in a mutant with green petioles and leaves. The non-functional *rap* gene was not able to complement Arabidopsis *tt19*, while wild-type *RAP* was successful [48].

In our analyses, all six independently generated white mutants of red varieties displayed the same deletion of a 4 bp repeat in *Bract1*, whereas the original varieties all contained a fully functional copy of the gene. In addition, co-segregation of the deletion with the white phenotype was observed in a segregated population of 190 progeny. Furthermore, a novel mutation leading to a homozygous recessive allele of *Bract1* among 184 samples obtained from irradiated cuttings of the heterozygous line SK183 led to a white phenotype (Table 3). Altogether, the results of this study present strong evidence that the four-base deletion in *Bract1* is the cause of the red-to-white shift in the poinsettia varieties analysed here. However, as anthocyanin biosynthesis involves several steps, other regulatory and structural genes might give rise to white mutants as well, as has been shown in numerous other examples [5, 39, 49, 52]. We did not detect these genes in our current plant material perhaps due to the much higher mutation rate of the *Bract1* gene than of less mutable genes.

**Table 3:** List of poinsettia varieties used in the present study

Variety ID	Variety name	Bract colour	Observation
1	Christmas Feelings	Red	
2	Christmas Glory	Red	
3	Joy	Red	
4	Bravo	Red	
5	Titan	Red	
6	SK130	Red	
7	Christmas Feelings Pearl	White	Mutation from Chr. Feelings
8	Christmas Glory White	White	Mutation from Chr. Glory
9	Joy White	White	Mutation from Joy
10	Bravo White	White	Mutation from Bravo
11	Titan White	White	Mutation from Titan
12	SK130 White	White	Mutation from SK130
13	Vintage	Red	
14	Christmas Aurora	Red	
15	Happy Day	Red	
16	Tabalunga	Red	
17	Christmas Day	Red	
18	Christmas Eve	Red	
19	Noel	Red	
20	Valentino	Red	
21	Prestige Red	Red	
22	Christmas Cracker	Red	

### *Bract1* contains a short highly mutable four-base repeat

Upon X-ray treatment, red poinsettia plants produce progeny bearing white phenotypes with high frequencies, often based on only 10 irradiated cuttings (von Tubeuf, Selecta One, pers. comm., Selecta One). This phenomenon is associated with a deletion in a short repeat in the *Bract1* gene of white mutants in a homozygous state. The mutations in all six independent mutant pairs that we detected are exactly identical, which indicates that the X-ray treatment did not directly cause the mutation but rather led to changes indirectly by stimulating the DNA repair mechanisms via replication errors, by increasing recombination or by the other mechanisms discussed for mutations in repeat sequences [56]. The possible involvement of replication-based errors is supported by our observation that upon amplification of the repeat via standard PCR from cloned *Bract1* wild-type or mutant allele, a low level of variants carrying four-base indels can always be detected (data not shown).

Radiation is frequently used as a tool for mutagenic breeding in poinsettia. In contrast to ethyl-methanesulphonate (EMS)-based chemical mutagenesis, which produces point mutations with high frequency [25], ionizing radiation (e.g., X-rays and γ-rays) induces DNA oxidative damage, such as double-strand breaks (DSBs), base substitutions, deletions and chromosomal alterations, at a lower frequency, frequently resulting in loss of gene function [35, 36, 51]. SSRs are among the most variable types of repetitive sequences in the genome [19]. Studies have shown that SSR instability increases with plant development [22] and abiotic stress [74]. This might be another explanation for the frequent observation of repeat changes in the *Bract1* gene after X-ray irradiation, although the small number of repeats (i.e., three) of four base pairs each does not fit the most widely applied criteria used to define SSRs, which usually focus on sequences with a larger number of repeats.

However, little information about the genetics and dynamics is available for short repeats. A majority of studies compared historical events for mostly shorter SSRs (2 and 3 bp repeats with larger repeat numbers) in present-day populations or the dynamic repeats responsible for human diseases (mostly trinucleotide repeats), which usually display effects beyond those of large numbers of repeats (> 30 repeats [56]).

Our observation that a large number of mutation events could be observed in the side shoots of ten irradiated plants indicates an unusually high mutation rate, which is in contrast to the few reports in which exact mutation rates have been reported for vegetatively propagated crops [65]. In one example, the woody ornamental plant *Tibouchina urvelliana* was irradiated three independent times with a 45 Gy dose, resulting in 0.06% dwarf mutants each time [65]. However, several authors reported that the radiosensitivity of vegetative tissues varies greatly among species and tissues [20], so exact comparative estimations of mutation frequencies have a very limited accuracy among species and conditions. However, experiments with transgenic Arabidopsis lines harbouring constructs designed to analyse restoration of GUS open reading frames by either recombination or by restoring in-frame translation by mutations in SSRs demonstrate the occurrence of easily detectable numbers of somatic mutation events [22, 74]. Together with the careful selection of side shoots after X-ray irradiation of poinsettia, this finding may explain the high rate of recessive mutations detected here.

In this study, we showed that the poinsettia *Bract1* gene is an active GST gene involved in the expression of anthocyanins in poinsettia bracts. Furthermore, a 4 bp deletion in a short repeat within the coding region of *Bract1* is the most likely cause of many mutations that lead to a white bract colour. This mutation occurs with an unusually high frequency and is presumably an indirect effect of X-ray mutagenesis. Future analyses using mutagenesis in transgenic *Arabidopsis* lines harbouring *Bract1* might help elucidate the causes of the high instability of this repeat. Moreover, this result might also serve as a reference for the study of other repeat-containing structural genes as potential mutational hot spots in plant genomes.

## Conclusions

In this study, we showed that the poinsettia *Bract1* gene is an active GST gene involved in the expression of anthocyanins in poinsettia bracts. Furthermore, a four base pair deletion in a short repeat within the coding region of *Bract1* is the most likely cause of many white mutations for bract colour. This mutation occurs with an unusually high frequency and is presumably an indirect effect of the x-ray mutagenesis. Future analyses using mutagenesis in transgenic Arabidopsis lines harbouring the *Bract1* might help to elucidate the causes of the high instability of this repeat. Moreover, it might also serve as an example for other repeat containing structural genes in plant genomes as potential mutational hot spots.

## Methods

### Plant material

A range of red- and white-bracted varieties of poinsettia was used in this study for the different analyses (Table 3). Among the varieties, we used so-called pairs of red varieties and white mutants generated from these varieties by γ-ray or X-ray mutagenesis by the company Selecta One (Stuttgart, Germany). In addition, a segregating population containing 190 progeny from the “Joy x Joy white” cross, one such pair, was also used for analysis. The plant material used are exclusively cultivated varieties of the species *Euphorbia pulcherrima* which were provided by the company Selecta One. Identification of the material was conducted by Dr. Robert Boehm and Mr. Guido von Tubeuf. There were no vouchers taken and deposited.

Leaf and bract samples for DNA and RNA isolation were harvested at Selecta One, immediately frozen in liquid nitrogen, shipped on dry ice and stored at − 80 °C at the Institute for Plant Genetics of the Leibniz Universität Hannover (Hannover, Germany). Moreover, rooted cuttings of three red (‘Aurora’, ‘Aurora Jingle’ and ‘SK183’) and one pink (‘SK159 Dark Pink’) variety were irradiated with γ-rays (20 Gy) and further developed, and new cuttings were generated by the company Selecta One. The progeny were used for fragment analysis (please refer to section “Poinsettia genotyping and fragment analysis”).

Seeds of the *Arabidopsis thaliana* Columbia (*col-0)* genotype were available at the Institute for Plant Genetics of the Leibniz Universität Hannover; these seeds were originally obtained from the Arabidopsis information service in Frankfurt, Germany [12], and subsequently propagated in isolated greenhouse compartments. Seeds of the mutant line *tt19–8* (stock number: CS2105587) were obtained from the Arabidopsis Biological Resource Center (ABRC). Seeds were sown in Einheitserde P substrate, and seedlings were placed in long-day conditions (16 h light/8 h dark, 22 °C) for 2 weeks to induce flowering. Seedlings of the wild-type and mutants analysed for the expression of anthocyanins were grown under supplemental red/blue LED light (GP LED production DR/B 120 LB, Philips, Germany) to stimulate anthocyanin development.

### DNA and RNA isolation

For the poinsettia samples, DNA was isolated from approximately 100 mg of leaf tissue using the NucleoSpin® Plant II Kit (Macherey–Nagel GmbH & Co., KG, Düren, Germany) according to the manufacturer’s instructions. Total RNA was isolated from approximately 100 mg of bract tissue using the mirPremier™ miRNA Isolation Kit (Sigma-Aldrich, St. Louis, USA). For Arabidopsis samples, total RNA was isolated from approximately 50 mg of leaf tissue using the Quick-RNA Plant Kit (Zymo Research, Irvine, USA). cDNA synthesis was performed using the FastGene Scriptase Basic cDNA Kit (Nippon Genetics Europe GmbH, Düren, Germany) according to the manufacturer’s recommendations. The DNA and total RNA concentrations and quality were assessed using a NanoDrop™ 2000 (Thermo Fisher Scientific, Wilmington, USA) and gel electrophoresis.

### GST expression by RT-qPCR

Two endogenous reference genes (Translation elongation factor 1 beta (*EF1B*) and Translation elongation factor 1 alpha (*EF1A*)) were used to normalize the *Bract1* expression data. Primer sequences are available in Additional File S4. The amplification efficiency for all primers was obtained from relative standard curves. Three independent biological replicates were used for each of the varieties. RT-qPCRs were performed using the qPCRBIO SyGreen Mix Lo-ROX Kit (Nippon Genetics Europe GmbH) according to the manufacturer’s recommendations. Briefly, reactions were carried out in technical triplicates in a volume of 10 μL containing 5 μL of qPCRBIO SyGreen Mix Lo-ROX, 10 μmol of gene-specific forward and reverse primers, and 4 μL of a 1:50 cDNA dilution. RT-qPCRs were performed using a StepOne™ Real-Time PCR System (Applied Biosystems, Singapore, Singapore). The normalized relative quantity (NRQ) was calculated according to the Pfaffl equations [59]. The real-time data for this study are provided according to the Minimum Information for Publication of Quantitative Real-Time PCR Experiments guidelines [6]. Pairwise statistical analysis between each red variety and its white counterpart was performed using the Relative Expression Software Tool (REST) v2.0.13 [60].

### *Bract1* gene sequencing

The poinsettia variety ‘Vintage’ was used for full-length sequencing of the GST-like gene (hereafter named *Bract1*). PCRs were performed in a 50 μL reaction containing 50 ng of DNA template, 1X PrimeSTAR® Buffer (Mg^2+^ plus), 0.2 mM each dNTP, 0.25 μM forward and reverse primers and 1.25 U of PrimeSTAR® HS DNA Polymerase (Takara Bio Inc., Kusatsu, Japan). The cycling conditions were 95 °C for 3 min; 30 cycles of 95 °C for 30 s, 60 °C for 30 s and 72 °C for 2 min; and a final extension of 10 min at 72 °C. The PCR products were resolved in a 1% (w/v) agarose gel by horizontal electrophoresis for 90 min at 100 V. The correct bands were excised from the gel and purified using the NucleoSpin® Gel and PCR Clean-up Kit (Macherey–Nagel) following the manufacturer’s recommendations. Finally, the purified PCR fragments were sent to Eurofins Genomics (Ebersberg, Germany) for Sanger sequencing. The generated sequences were aligned using the ClustalW function in the BioEdit Sequence Alignment Editor v7.2.5 [30], and a final full-length gene sequence for *Bract1* was generated.

The coding sequences (CDSs) of *Bract1* from 14 red- and white-bracted poinsettia varieties (‘Noel’, ‘Valentino’, ‘Christmas Feelings’, ‘Christmas Feelings Pearl’, ‘Christmas Glory’, ‘Christmas Glory White’, ‘Joy’, ‘Joy White’, ‘Titan’, ‘Titan White’, ‘Bravo’, ‘Bravo White’, ‘SK130’ and ‘SK130 White’) were further sequenced. PCRs were performed in a 50 μL reaction containing 1 μL of undiluted cDNA, 1X PrimeSTAR® Buffer (Mg^2+^ plus), 0.2 mM each dNTP, 0.25 μM forward and reverse primers and 1.25 U of PrimeSTAR® HS DNA Polymerase (Takara). The cycling conditions were 95 °C for 3 min; 30 cycles of 95 °C for 30 s, 60 °C for 30 s and 72 °C for 45 s; and a final extension of 10 min at 72 °C. The sequencing strategy was the same as that used for the full-length sequencing analysis. The generated sequences were aligned using the ClustalW function in the BioEdit Sequence Alignment Editor v7.2.5. Primer sequences are available in Additional File S4.

### Poinsettia genotyping and fragment analysis

To detect changes in the repeat structure of the *Bract1* gene, a genotyping approach based on the fluorescent labelling of PCR fragments [64] was applied. DNA samples were PCR amplified in a 20 μL reaction containing 50 ng of DNA template, 1X Williams buffer, 0.15 mM each dNTP, 0.0125 μM forward primer, 0.07 μM universal FAM-labelled M13 primer, 0.25 μM reverse primer and 1 U of DCSPol DNA Polymerase (DNA Cloning Service, Hamburg, Germany). The cycling conditions were 94 °C for 3 min; 24 cycles of 94 °C for 45 s, 59 °C for 1 min and 72 °C for 1 min; 6 cycles of 94 °C for 30 s, 52 °C for 45 s and 72 °C for 1 min; and a final extension of 10 min at 72 °C. Fifty microliters of formamide loading dye (98% formamide, 10 mM EDTA, 0.05% pararosanilin) was added to each reaction, which was then incubated at 95 °C for 5 min. The PCR products were resolved in a 6% (w/v) acrylamide gel via vertical electrophoresis using a LI-COR Gene Readir 4200 DNA Analyser (LI-COR Biosciences, Nebraska, USA). The varieties from Table 3 and the progeny of the irradiated variety ‘Aurora’ were genotyped using fluorescent labelling of PCR fragments. The progeny of the irradiated varieties ‘Aurora Jingle’ and ‘SK159 Dark Pink’ were analysed by capillary electrophoresis on an ABI 3730 XL system at Microsynth AG (Balgach, Switzerland). Primer sequences are available in Additional File S4.

### Phylogenetic analysis

Protein sequences for *Bract1* and 95 different poinsettia GSTs, retrieved from our previous study [72], were predicted with TransDecoder [29] and used for the construction of a phylogenetic tree. Moreover, protein sequences for known anthocyanin-related GSTs from other species were included in the analysis: *CkmGST3* (*Cyclamen persicum* x *Cyclamen purpurascens*, GenBank - AB682678.1), *LcGST4* (*Litchi chinensis*, GenBank - KT946768.1), *VvGST4* (*Vitis vinifera*, GenBank - AY971515.1), *PhAN9* (*Petunia hybrida*, GenBank - Y07721.1), *PpRiant1* (*Prunus persica*, GenBank - KT312847.1), *PpRiant2* (*P. persica*, GenBank - KT312848.1), *AtGSTF11* (*Arabidopsis thaliana*, GenBank - NM_111189.3) and *AtTT19* (*A. thaliana*, GenBank - NM_121728.4). The putative protein sequences of all the GSTs are available in Additional File S5.

Sequence alignment was performed using MUSCLE [17], and the phylogenetic tree was constructed with MEGA X v10.0.5 [41] using the maximum likelihood (ML) method with the Whelan and Goldman matrix-based model using a discrete gamma distribution (WAG+G) [73]. The best model was estimated using MEGAX. The tree topology was tested via a bootstrap analysis with 1000 replicates. For better visualization of the phylogenetic tree, Tree Of Life (iTOL) software, version 4.2.3 [44] (https://itol.embl.de/), was used.

### Plasmid construction and *Agrobacterium*-mediated infiltration

The coding sequence of *Bract1* was amplified from the poinsettia varieties ‘Vintage’ and ‘Christmas Feelings Pearl’ to capture both wild-type and mutated alleles. The primers used for amplification are available in Additional File S4. The PCR fragments were inserted in the sense orientation into the *BamHI*-*HindIII* site of the C757pGFPU10–35 s-ocs-LH (Additional File S6) binary vector (DNA Cloning Service, Hamburg, Germany) using the In-Fusion® HD Cloning Kit (Takara Bio Inc., Kusatsu, Japan). The vector contains a GFP gene under the control of the Arabidopsis ubiquitin promoter (*ProAtUbi::GFP*) and a 35S promoter upstream of the multiple cloning site into which inserts were cloned. The final expression vectors contained either the wild-type allele (*35S::Bract1*) or the mutated allele (*35S::Bract1_mut*). The vector containing only GFP was also used for transformation as a negative control (only *ProAtUbi::GFP*).

The expression vectors were introduced into *Agrobacterium tumefaciens* strain GV3101 via electroporation. Transformation of the *A. thaliana* mutant line *tt19–8* was performed using the floral dip method [11, 75]. For transgenic plant selection, T_0_ seeds were sown in soil, and GFP-expressing seedlings were selected to produce T_1_ and subsequently T_2_ progeny to achieve *GST* homozygosity. T_2_ seedlings of *tt19*/*35S::Bract1* and *tt19*/*35S::Bract1_mut* transgenic plants were used for phenotypic analysis. Non-transformed *tt19–8*, *Col-0* and *tt19*/*ProAtUbi::GFP* seedlings were used as controls. Seedlings were placed under red light to stimulate anthocyanin biosynthesis. To confirm correct *GST* integration, 10 independent biological replicates, each representing an independently selected transgenic line from the T_2_ progeny of *tt19*/*35S::Bract1* and *tt19*/*35S::Bract1_mut* transgenic plants, were used for GST genotyping. The protocol was the same as that used in the section *“Poinsettia genotyping and fragment analysis”*.

### Analysis of the GST repeat

To understand the origin of the microsatellite-like repeat in the *Bract1* gene, orthologous GST genes from Euphorbiaceae species were retrieved by BLASTN against the Euphorbiaceae (taxid: 3977) nucleotide database. GST-like genes from the Euphorbiaceae species *Ricinus communis* (GenBank - XM_002532928.3), *Manihot esculenta* (GenBank - XM_021748071.1), *Jatropha curcas* (GenBank - XM_012219312.2), *Hevea brasiliensis* (GenBank - XM_021787187.1), *Euphorbia esula* (GenBank - PJAE01736713.1) and *Euphorbia pekinensis* [7, 72], as well the anthocyanin-related GSTs *CkmGST3* (*C. persicum* x *C. purpurascens*, GenBank - AB682678.1), *LcGST4* (*L. chinensis*, GenBank - KT946768.1), *VvGST4* (*V. vinifera*, GenBank - AY971515.1), *PhAN9* (*P. hybrida*, GenBank - Y07721.1), *PpRiant1* (*P. persica*, GenBank - KT312847.1), *PpRiant2* (*P. persica*, GenBank - KT312848.1) and *AtTT19* (*A. thaliana*, GenBank - NM_121728.4) were used to construct a phylogenetic tree.

Sequence alignment was performed using MUSCLE [17], and the phylogenetic tree was constructed with MEGA X v10.0.5 [41] using the ML method with the Tamura 3-parameter matrix-based model [68] using a discrete gamma distribution with invariant sites (T92 + G + I). The best model was estimated using MEGAX. The tree topology was tested via a bootstrap analysis with 1000 replicates. For better visualization of the phylogenetic tree, Tree Of Life (iTOL) software, version 4.2.3 [44] (https://itol.embl.de/), was used.

## Supplementary Information

**Additional file 1 **Full-length sequence of the *Bract1* gene, CDS sequences of red and white poinsettia varieties, full sequence and truncated version of the BRACT1 protein.

**Additional file 2.** Full length gel images referent to Fig. 3a, Fig. 3b and Fig. 6a from this publication.

**Additional file 3 **Sequence similarity of *Bract1* with other anthocyanin-related GSTs.

**Additional file 4.** List of primers used in each of the analyses in the present study.

**Additional file 5 **Deducted protein sequences from the *Bract1* and 95 GSTs from *E. pulcherrima* GSTs, as well as anthocyanin-related GSTs from other species.

**Additional file 6 **Schematic representation from the C757pGFPU10–35 s-ocs-LH binary vector used for the constructions of transformation plasmids containing either the wild-type allele (*35S::Bract1*) or the mutated allele (*35S::Bract1_mut*) from the poinsettia *GST*.

**Additional file 7 **Sequence alignment of *Bract1*, anthocyanin-related GSTs and orthologue GSTs from Euphorbiaceae species.

## Data Availability

All data generated or analysed during this study are included in this published article and its supplementary information files. DNA sequence from the *Bract1* gene is available in the GenBank repository under accession number MW718861. The CDS sequences from several poinsettia genotypes generated during the current study are available in the GenBank repository under accession numbers MW718847 - MW718860.

## Abbreviations

ABC: ATP binding cassette transporter
CaMV: Cauliflower mosaic virus
cDNA: Complementary DNA
CDS: Coding sequence
DNA: Deoxyribonucleic acid
DSB: Double-strand break
EMS: ethyl-methanesulphonate
ER: Endoplasmic reticulum
GFP: Green fluorescent protein
GST: Glutathione S-transferase
GUS: β-glucuronidase protein
Gy: Gray (unit of ionizing radiation)
Indel: Insertion and deletion variations
kDA: Kilo Dalton
MATE: Multi-antimicrobial extrusion protein
MBW complex: MYB-bHLH-WD40 complex
mRNA: Messenger RNA
NRQ: Normalized relative quantity
PCR: Polymerase chain reaction
RNA: Ribonucleic acid
RT-qPCR: Quantitative reverse transcription PCR
SNP: Single nucleotide polymorphism
SSR: Single sequence repeat
tt19: Transparent testa 19 Arabidopsis mutant
