# Supplementary material for: A highly mutable GST is essential for bract colouration in Euphorbia pulcherrima Willd. Ex Klotsch
Source: BMC Genomics. 2021 Mar 23;22:208. doi: 10.1186/s12864-021-07527-z (PMC7988969; doi:10.1186/s12864-021-07527-z)
Supplement: Supplementary file 1 — Additional file 1 Full-length sequence of the Bract1 gene, CDS sequences of red and white poinsettia varieties, full sequence and truncated version of the BRACT1 protein. [file 12864_2021_7527_MOESM1_ESM.docx]

**Additional File S1. Full-length sequence of the *Bract1* gene, CDS sequences of red and white poinsettia varieties, full sequence and truncated version of the BRACT1 protein.**

>Bract1_full_length_gene

ATGGTAGTGAAAGTGTATGGAGCAGCTCAGGCAGCTTGCCCACAAAGAGTAATGGCCTGCCTTTTAGAGAAAGATATTCCTTTTGATCTTGTTCATGTTGATCTTCCTTCTGCTCAACATAAACTCTCTTCCTTCCTTCTCAAACAGGTTCTTTACTTCCCTTTTTTTACTATACATTTCTTCTAGGCCTAATATACATCTAGACCCCCTATAGTTGTTCCTGAAAACCCCTCAGCCCCCTGAACTTGTTAAAGTGGATCTTACAGCCCCTTAAACTTGGTCAAACTGAACCTCAAAACCCCTTAATGGTAACATGCCCAGTTTTGTTCCGGTAAATCAGTTTTCGATTCAATCTTTGACGAAACAATCTTAGATTCAATCTTTAGAAAAATAATACTTGACATATCAGCATCAAGCGGCTATGGGGTTCACTTTTAACAAGTTCAGGGTGCTATAAAGTCCACTTTAATAAATTCAGGGTTAAGGATACCTTTTGTAAGTTTAGGTGGTTGAGGGGTTTTAAGGGAACTATAGGAGGCCTAGATTTATTAGCTCTCTTGTTTGATGTCACAATTAATTATGTTTATTTATTTATTCTGTAGCCCTTTGGGTTAGTTCCAGCTATAGAAGATGGGGATTTCAGGCTTTTTGGTATGTTTCTTAATCTTTTCATTTCAGTGATAGCCCTCAGTATTTCGTTTTACTAAGATTTCGGGACCACAATTCGTTTTTGTAGTGTCACTATGGATAATTGTTAAAAATGAGACATGTACAAAACAAATCCTATTCGAATCTTCTATCGTTGGTCATAACCACATGGATATCTACGCATAAAAATACCAAAAAATATTTTAACATGTACATGTTCTTTTGTATTCCGTCCACTATTTCGATAAGCATCGATTTATCGTTTTTTGGCTTTAAATTATAATGGACTAAACTAAAATGATTCATTAAACTGTCTGAACTTCTTTATTGTGAAATCACGGATAGCTGTTGCAACTCCACTTCAGGGTCAATTGGAAACAACATCTCTGTAATTACATGGGTAAGGCTGCATACACTCAACCCCCCGACACTGCTTGTGAGGGAGCCTTATTAGGCATTGGGGTGATGTTGTTGTTTTACACGTTATTTTGTATCATTGTAATCTATCAAACTATTATAATTACTTATTACCAAGTATAATTTATTACATTGATTAAAGTATAATTTATTCCACTAAATTTATGTTTTATGCACCTTACCCTGAACTTTGATTTTTTATTTAGTATTATAAAATGTTGTTTAAGTAAATAAAATAGATACTATTTAAAAATAATTTAGAAAAAAATAATAAAATAGAGCAAGGTCTCCATACAAAACCATCGTACTTATTTGAACAGATATATATGTAGTGTTATTCATATTTTTTTTTATAATAACATAGAACTGATGAATCTGGATTAGAAATGATGATATAATGGCTTGCCTCATTCACGATCACACAATTGATAAGTCTGATTTTACCAACAAATATCAGTTTTTCAATATTATGTGTTGCTATTTTCTTGAAGAAGAAATTTTGCACGACCATATTTAAGAATAGGCTTAGATTGATCGGTCAAACAAAATCTTAGGTTATTTTTTCATTTTCCTTTTCTCATTAGTTAGAATCAAAATTTGGAATTAAATTTTTTGTTTTAATTTTACTTCTAACTATTGAGATCATATATCACCAAATATATGCTTTCTTTATTATTTCTACATAAAAAAATATATGGTTTTACAACTACCTAACTATGCTTTTTTTTAAAGTTTGCCTCATTAGGCTTACAACTACCAAACTATGTTGAATTAATATAATTTTGTTTGTGTGTGTGAAGAATCAAGAGCCATAATGAGATACTATGCAACAAAATATGAAGAAAGAGGGCCCAATTTGTTAGGAAAAACATTAGAAGAGAAAGCAATAGTTGATCAATGGGTTGAAGTGGAAGCCCATAATTTCAATAATTTGGTTTACAATATTGTAATTGAAGTTTTGATAAAGCCAAAAATGGGGGAACAAGGTGACATCAACATAGTCAAAAGCTGTGAACATAAGCTGGATAAAGTGTTCGATGTGTACGAGGAAAGGCTATCCAGTTCCAAATATCTTGGAGGAGATTATTTCACACTTGCTGATTTAACCCATATGCCTTCCATTAGGTACCTTGTTCATGAGCTTGGGTTAGCCCATTTGGTTCACAATAGAAACAAGGTCAATGCTTGGTGGATTGATATATCGGACCGACCGGCTTGGAAAAATTTGATGATTCTTGCTGGTTATTAG

>Bract1_CDS

ATGGTAGTGAAAGTGTATGGAGCAGCTCAGGCAGCTTGCCCACAAAGAGTAATGGCCTGCCTTTTAGAGAAAGATATTCCTTTTGATCTTGTTCATGTTGATCTTCCTTCTGCTCAACATAAACTCTCTTCCTTCCTTCTCAAACAGCCCTTTGGGTTAGTTCCAGCTATAGAAGATGGGGATTTCAGGCTTTTTGAATCAAGAGCCATAATGAGATACTATGCAACAAAATATGAAGAAAGAGGGCCCAATTTGTTAGGAAAAACATTAGAAGAGAAAGCAATAGTTGATCAATGGGTTGAAGTGGAAGCCCATAATTTCAATAATTTGGTTTACAATATTGTAATTGAAGTTTTGATAAAGCCAAAAATGGGGGAACAAGGTGACATCAACATAGTCAAAAGCTGTGAACATAAGCTGGATAAAGTGTTCGATGTGTACGAGGAAAGGCTATCCAGTTCCAAATATCTTGGAGGAGATTATTTCACACTTGCTGATTTAACCCATATGCCTTCCATTAGGTACCTTGTTCATGAGCTTGGGTTAGCCCATTTGGTTCACAATAGAAACAAGGTCAATGCTTGGTGGATTGATATATCGGACCGACCGGCTTGGAAAAATTTGATGATTCTTGCTGGTTATTAG

>Noel_CDS

ATGGTAGTGAAAGTGTATGGAGCAGCTCAGGCAGCTTGCCCACAAAGAGTAATGGCCTGCCTTTTAGAGAAAGATATTCCTTTTGATCTTGTTCATGTTGATCTTCCTTCTGCTCAACATAAACTCTCTTCCTTCCTTCTCAAACAGCCCTTTGGGTTAGTTCCAGCTATAGAAGATGGGGATTTCAGGCTTTTTGAATCAAGAGCCATAATGAGATACTATGCAACAAAATATGAAGAAAGAGGGCCCAATTTGTTAGGAAAAACATTAGAAGAGAAAGCAATAGTTGATCAATGGGTTGAAGTGGAAGCCCATAATTTCAATAATTTGGTTTACAATATTGTAATTGAAGTTTTGATAAAGCCAAAAATGGGGGAACAAGGTGACATCAACATAGTCAAAAGCTGTGAACATAAGCTGGATAAAGTGTTCGATGTGTACGAGGAAAGGCTATCCAGTTCCAAATATCTTGGAGGAGATTATTTCACACTTGCTGATTTAACCCATATGCCTTCCATTAGGTACCTTGTTCATGAGCTTGGGTTAGCCCATTTGGTTCACAATAGAAACAAGGTCAATGCTTGGTGGATTGATATATCGGACCGACCGGCTTGGAAAAATTTGATGATTCTTGCTGGTTATTAG

>Valentino_CDS

ATGGTAGTGAAAGTGTATGGAGCAGCTCAGGCAGCTTGCCCACAAAGAGTAATGGCCTGCCTTTTAGAGAAAGATATTCCTTTTGATCTTGTTCATGTTGATCTTCCTTCTGCTCAACATAAACTCTCTTCCTTCCTTCTCAAACAGCCCTTTGGGTTAGTTCCAGCTATAGAAGATGGGGATTTCAGGCTTTTTGAATCAAGAGCCATAATGAGATACTATGCAACAAAATATGAAGAAAGAGGGCCCAATTTGTTAGGAAAAACATTAGAAGAGAAAGCAATAGTTGATCAATGGGTTGAAGTGGAAGCCCATAATTTCAATAATTTGGTTTACAATATTGTAATTGAAGTTTTGATAAAGCCAAAAATGGGGGAACAAGGTGACATCAACATAGTCAAAAGCTGTGAACATAAGCTGGATAAAGTGTTCGATGTGTACGAGGAAAGGCTATCCAGTTCCAAATATCTTGGAGGAGATTATTTCACACTTGCTGATTTAACCCATATGCCTTCCATTAGGTACCTTGTTCATGAGCTTGGGTTAGCCCATTTGGTTCACAATAGAAACAAGGTCAATGCTTGGTGGATTGATATATCGGACCGACCGGCTTGGAAAAATTTGATGATTCTTGCTGGTTATTAG

>Christmas_Feelings_CDS

ATGGTAGTGAAAGTGTATGGAGCAGCTCAGGCAGCTTGCCCACAAAGAGTAATGGCCTGCCTTTTAGAGAAAGATATTCCTTTTGATCTTGTTCATGTTGATCTTCCTTCTGCTCAACATAAACTCTCTTCCTTCCTTCTCAAACAGCCCTTTGGGTTAGTTCCAGCTATAGAAGATGGGGATTTCAGGCTTTTTGAATCAAGAGCCATAATGAGATACTATGCAACAAAATATGAAGAAAGAGGGCCCAATTTGTTAGGAAAAACATTAGAAGAGAAAGCAATAGTTGATCAATGGGTTGAAGTGGAAGCCCATAATTTCAATAATTTGGTTTACAATATTGTAATTGAAGTTTTGATAAAGCCAAAAATGGGGGAACAAGGTGACATCAACATAGTCAAAAGCTGTGAACATAAGCTGGATAAAGTGTTCGATGTGTACGAGGAAAGGCTATCCAGTTCCAAATATCTTGGAGGAGATTATTTCACACTTGCTGATTTAACCCATATGCCTTCCATTAGGTACCTTGTTCATGAGCTTGGGTTAGCCCATTTGGTTCACAATAGAAACAAGGTCAATGCTTGGTGGATTGATATATCGGACCGACCGGCTTGGAAAAATTTGATGATTCTTGCTGGTTATTAG

>Christmas_Glory_CDS

ATGGTAGTGAAAGTGTATGGAGCAGCTCAGGCAGCTTGCCCACAAAGAGTAATGGCCTGCCTTTTAGAGAAAGATATTCCTTTTGATCTAGTTCATGTTGATCTTCCTTCTGCTCAACACAAACTCTCTTCCTTCCTTCTCAAACAGCCATTTGGGTTAGTTCCAGCTATAGAAGATGGGGATTTCAGGCTTTTTGAATCAAGAGCCATAATGAGATACTATGCAACAAAATATGAAGAAAGAGGGCCCAATTTGTTAGGAAAAACATTAGAAGAGAAAGCAATAGTTGATCAATGGGTTGAAGTGGAAGCCCATAATTTCAATAATTTGGTTTACAATATTGTAATTGAAGTTTTGATAAAGCCAAAAATGGGGGAACAAGGTGACATCAACATAGTCAAAAGCTGTGAACATAAGCTGGATAAAGTGTTCGATGTGTACGAGGAAAGGCTATCCAGTTCCAAATATCTTGGAGGAGATTATTTCACACTTGCTGATTTAACCCATATGCCTTCCATTAGGTATCTTGTTCATGAGCTTGGGTTAGCCCATTTGGTTCACAATAGAAACAAGGTCAGTGCTTGGTGGATTGATATATCGGACAGACCGGCTTGGAAAAATTTGATGATTCTTGCTGGTTATTAG

>Joy_CDS

ATGGTAGTGAAAGTGTATGGAGCAGCTCAGGCAGCTTGCCCACAAAGAGTAATGGCCTGCCTTTTAGAGAAAGATATTCCTTTTGATCTTGTTCATGTTGATCTTCCTTCTGCTCAACATAAACTCTCTTCCTTCCTTCTCAAACAGCCCTTTGGGTTAGTTCCAGCTATAGAAGATGGGGATTTCAGGCTTTTTGAATCAAGAGCCATAATGAGATACTATGCAACAAAATATGAAGAAAGAGGGCCCAATTTGTTAGGAAAAACATTAGAAGAGAAAGCAATAGTTGATCAATGGGTTGAAGTGGAAGCCCATAATTTCAATAATTTGGTTTACAATATTGTAATTGAAGTTTTGATAAAGCCAAAAATGGGGGAACAAGGTGACATCAACATAGTCAAAAGCTGTGAACATAAGCTGGATAAAGTGTTCGATGTGTACGAGGAAAGGCTATCCAGTTCCAAATATCTTGGAGGAGATTATTTCACACTTGCTGATTTAACCCATATGCCTTCCATTAGGTACCTTGTTCATGAGCTTGGGTTAGCCCATTTGGTTCACAATAGAAACAAGGTCAATGCTTGGTGGATTGATATATCGGACCGACCGGCTTGGAAAAATTTGATGATTCTTGCTGGTTATTAG

>Titan_CDS

ATGGTAGTGAAAGTGTATGGAGCAGCTCAGGCAGCTTGCCCACAAAGAGTAATGGCCTGCCTTTTAGAGAAAGATATTCCTTTTGATCTTGTTCATGTTGATCTTCCTTCTGCTCAACATAAACTCTCTTCCTTCCTTCTCAAACAGCCCTTTGGGTTAGTTCCAGCTATAGAAGATGGGGATTTCAGGCTTTTTGAATCAAGAGCCATAATGAGATACTATGCAACAAAATATGAAGAAAGAGGGCCCAATTTGTTAGGAAAAACATTAGAAGAGAAAGCAATAGTTGATCAATGGGTTGAAGTGGAAGCCCATAATTTCAATAATTTGGTTTACAATATTGTAATTGAAGTTTTGATAAAGCCAAAAATGGGGGAACAAGGTGACATCAACATAGTCAAAAGCTGTGAACATAAGCTGGATAAAGTGTTCGATGTGTACGAGGAAAGGCTATCCAGTTCCAAATATCTTGGAGGAGATTATTTCACACTTGCTGATTTAACCCATATGCCTTCCATTAGGTACCTTGTTCATGAGCTTGGGTTAGCCCATTTGGTTCACAATAGAAACAAGGTCAATGCTTGGTGGATTGATATATCGGACCGACCGGCTTGGAAAAATTTGATGATTCTTGCTGGTTATTAG

>Bravo_CDS

ATGGTAGTGAAAGTGTATGGAGCAGCTCAGGCAGCTTGCCCACAAAGAGTAATGGCCTGCCTTTTAGAGAAAGATATTCCTTTTGATCTAGTTCATGTTGATCTTCCTTCTGCTCAACACAAACTCTCTTCCTTCCTTCTCAAACAGCCATTTGGGTTAGTTCCAGCTATAGAAGATGGGGATTTCAGGCTTTTTGAATCAAGAGCCATAATGAGATACTATGCAACAAAATATGAAGAAAGAGGGCCCAATTTGTTAGGAAAAACATTAGAAGAGAAAGCAATAGTTGATCAATGGGTTGAAGTGGAAGCCCATAATTTCAATAATTTGGTTTACAATATTGTAATTGAAGTTTTGATAAAGCCAAAAATGGGGGAACAAGGTGACATCAACATAGTCAAAAGCTGTGAACATAAGCTGGATAAAGTGTTCGATGTGTACGAGGAAAGGCTATCCAGTTCCAAATATCTTGGAGGAGATTATTTCACACTTGCTGATTTAACCCATATGCCTTCCATTAGGTATCTTGTTCATGAGCTTGGGTTAGCCCATTTGGTTCACAATAGAAACAAGGTCAGTGCTTGGTGGATTGATATATCGGACAGACCGGCTTGGAAAAATTTGATGATTCTTGCTGGTTATTAG

>SK130_CDS

ATGGTAGTGAAAGTGTATGGAGCAGCTCAGGCAGCTTGCCCACAAAGAGTAATGGCCTGCCTTTTAGAGAAAGATATTCCTTTTGATCTTGTTCATGTTGATCTTCCTTCTGCTCAACATAAACTCTCTTCCTTCCTTCTCAAACAGCCCTTTGGGTTAGTTCCAGCTATAGAAGATGGGGATTTCAGGCTTTTTGAATCAAGAGCCATAATGAGATACTATGCAACAAAATATGAAGAAAGAGGGCCCAATTTGTTAGGAAAAACATTAGAAGAGAAAGCAATAGTTGATCAATGGGTTGAAGTGGAAGCCCATAATTTCAATAATTTGGTTTACAATATTGTAATTGAAGTTTTGATAAAGCCAAAAATGGGGGAACAAGGTGACATCAACATAGTCAAAAGCTGTGAACATAAGCTGGATAAAGTGTTCGATGTGTACGAGGAAAGGCTATCCAGTTCCAAATATCTTGGAGGAGATTATTTCACACTTGCTGATTTAACCCATATGCCTTCCATTAGGTACCTTGTTCATGAGCTTGGGTTAGCCCATTTGGTTCACAATAGAAACAAGGTCAATGCTTGGTGGATTGATATATCGGACCGACCGGCTTGGAAAAATTTGATGATTCTTGCTGGTTATTAG

>Christmas_Feelings_Pearl_CDS

ATGGTAGTGAAAGTGTATGGAGCAGCTCAGGCAGCTTGCCCACAAAGAGTAATGGCCTGCCTTTTAGAGAAAGATATTCCTTTTGATCTTGTTCATGTTGATCTTCCTTCTGCTCAACATAAACTCTCTTCCTTCTCAAACAGCCCTTTGGGTTAGTTCCAGCTATAGAAGATGGGGATTTCAGGCTTTTTGAATCAAGAGCCATAATGAGATACTATGCAACAAAATATGAAGAAAGAGGGCCCAATTTGTTAGGAAAAACATTAGAAGAGAAAGCAATAGTTGATCAATGGGTTGAAGTGGAAGCCCATAATTTCAATAATTTGGTTTACAATATTGTAATTGAAGTTTTGATAAAGCCAAAAATGGGGGAACAAGGTGACATCAACATAGTCAAAAGCTGTGAACATAAGCTGGATAAAGTGTTCGATGTGTACGAGGAAAGGCTATCCAGTTCCAAATATCTTGGAGGAGATTATTTCACACTTGCTGATTTAACCCATATGCCTTCCATTAGGTACCTTGTTCATGAGCTTGGGTTAGCCCATTTGGTTCACAATAGAAACAAGGTCAATGCTTGGTGGATTGATATATCGGACCGACCGGCTTGGAAAAATTTGATGATTCTTGCTGGTTATTAG

>Christmas_Glory_White_CDS

ATGGTAGTGAAAGTGTATGGAGCAGCTCAGGCAGCTTGCCCACAAAGAGTAATGGCCTGCCTTTTAGAGAAAGATATTCCTTTTGATCTTGTTCATGTTGATCTTCCTTCTGCTCAACATAAACTCTCTTCCTTCTCAAACAGCCCTTTGGGTTAGTTCCAGCTATAGAAGATGGGGATTTCAGGCTTTTTGAATCAAGAGCCATAATGAGATACTATGCAACAAAATATGAAGAAAGAGGGCCCAATTTGTTAGGAAAAACATTAGAAGAGAAAGCAATAGTTGATCAATGGGTTGAAGTGGAAGCCCATAATTTCAATAATTTGGTTTACAATATTGTAATTGAAGTTTTGATAAAGCCAAAAATGGGGGAACAAGGTGACATCAACATAGTCAAAAGCTGTGAACATAAGCTGGATAAAGTGTTCGATGTGTACGAGGAAAGGCTATCCAGTTCCAAATATCTTGGAGGAGATTATTTCACACTTGCTGATTTAACCCATATGCCTTCCATTAGGTACCTTGTTCATGAGCTTGGGTTAGCCCATTTGGTTCACAATAGAAACAAGGTCAATGCTTGGTGGATTGATATATCGGACCGACCGGCTTGGAAAAATTTGATGATTCTTGCTGGTTATTAG

>Joy_White_CDS

ATGGTAGTGAAAGTGTATGGAGCAGCTCAGGCAGCTTGCCCACAAAGAGTAATGGCCTGCCTTTTAGAGAAAGATATTCCTTTTGATCTTGTTCATGTTGATCTTCCTTCTGCTCAACATAAACTCTCTTCCTTCTCAAACAGCCCTTTGGGTTAGTTCCAGCTATAGAAGATGGGGATTTCAGGCTTTTTGAATCAAGAGCCATAATGAGATACTATGCAACAAAATATGAAGAAAGAGGGCCCAATTTGTTAGGAAAAACATTAGAAGAGAAAGCAATAGTTGATCAATGGGTTGAAGTGGAAGCCCATAATTTCAATAATTTGGTTTACAATATTGTAATTGAAGTTTTGATAAAGCCAAAAATGGGGGAACAAGGTGACATCAACATAGTCAAAAGCTGTGAACATAAGCTGGATAAAGTGTTCGATGTGTACGAGGAAAGGCTATCCAGTTCCAAATATCTTGGAGGAGATTATTTCACACTTGCTGATTTAACCCATATGCCTTCCATTAGGTACCTTGTTCATGAGCTTGGGTTAGCCCATTTGGTTCACAATAGAAACAAGGTCAATGCTTGGTGGATTGATATATCGGACCGACCGGCTTGGAAAAATTTGATGATTCTTGCTGGTTATTAG

>Titan_White_CDS

ATGGTAGTGAAAGTGTATGGAGCAGCTCAGGCAGCTTGCCCACAAAGAGTAATGGCCTGCCTTTTAGAGAAAGATATTCCTTTTGATCTTGTTCATGTTGATCTTCCTTCTGCTCAACATAAACTCTCTTCCTTCTCAAACAGCCCTTTGGGTTAGTTCCAGCTATAGAAGATGGGGATTTCAGGCTTTTTGAATCAAGAGCCATAATGAGATACTATGCAACAAAATATGAAGAAAGAGGGCCCAATTTGTTAGGAAAAACATTAGAAGAGAAAGCAATAGTTGATCAATGGGTTGAAGTGGAAGCCCATAATTTCAATAATTTGGTTTACAATATTGTAATTGAAGTTTTGATAAAGCCAAAAATGGGGGAACAAGGTGACATCAACATAGTCAAAAGCTGTGAACATAAGCTGGATAAAGTGTTCGATGTGTACGAGGAAAGGCTATCCAGTTCCAAATATCTTGGAGGAGATTATTTCACACTTGCTGATTTAACCCATATGCCTTCCATTAGGTACCTTGTTCATGAGCTTGGGTTAGCCCATTTGGTTCACAATAGAAACAAGGTCAATGCTTGGTGGATTGATATATCGGACCGACCGGCTTGGAAAAATTTGATGATTCTTGCTGGTTATTAG

>Bravo_White_CDS

ATGGTAGTGAAAGTGTATGGAGCAGCTCAGGCAGCTTGCCCACAAAGAGTAATGGCCTGCCTTTTAGAGAAAGATATTCCTTTTGATCTTGTTCATGTTGATCTTCCTTCTGCTCAACATAAACTCTCTTCCTTCTCAAACAGCCCTTTGGGTTAGTTCCAGCTATAGAAGATGGGGATTTCAGGCTTTTTGAATCAAGAGCCATAATGAGATACTATGCAACAAAATATGAAGAAAGAGGGCCCAATTTGTTAGGAAAAACATTAGAAGAGAAAGCAATAGTTGATCAATGGGTTGAAGTGGAAGCCCATAATTTCAATAATTTGGTTTACAATATTGTAATTGAAGTTTTGATAAAGCCAAAAATGGGGGAACAAGGTGACATCAACATAGTCAAAAGCTGTGAACATAAGCTGGATAAAGTGTTCGATGTGTACGAGGAAAGGCTATCCAGTTCCAAATATCTTGGAGGAGATTATTTCACACTTGCTGATTTAACCCATATGCCTTCCATTAGGTACCTTGTTCATGAGCTTGGGTTAGCCCATTTGGTTCACAATAGAAACAAGGTCAATGCTTGGTGGATTGATATATCGGACCGACCGGCTTGGAAAAATTTGATGATTCTTGCTGGTTATTAG

>SK130_White_CDS

ATGGTAGTGAAAGTGTATGGAGCAGCTCAGGCAGCTTGCCCACAAAGAGTAATGGCCTGCCTTTTAGAGAAAGATATTCCTTTTGATCTTGTTCATGTTGATCTTCCTTCTGCTCAACATAAACTCTCTTCCTTCTCAAACAGCCCTTTGGGTTAGTTCCAGCTATAGAAGATGGGGATTTCAGGCTTTTTGAATCAAGAGCCATAATGAGATACTATGCAACAAAATATGAAGAAAGAGGGCCCAATTTGTTAGGAAAAACATTAGAAGAGAAAGCAATAGTTGATCAATGGGTTGAAGTGGAAGCCCATAATTTCAATAATTTGGTTTACAATATTGTAATTGAAGTTTTGATAAAGCCAAAAATGGGGGAACAAGGTGACATCAACATAGTCAAAAGCTGTGAACATAAGCTGGATAAAGTGTTCGATGTGTACGAGGAAAGGCTATCCAGTTCCAAATATCTTGGAGGAGATTATTTCACACTTGCTGATTTAACCCATATGCCTTCCATTAGGTACCTTGTTCATGAGCTTGGGTTAGCCCATTTGGTTCACAATAGAAACAAGGTCAATGCTTGGTGGATTGATATATCGGACCGACCGGCTTGGAAAAATTTGATGATTCTTGCTGGTTATTAG

>Bract1 – *Euphorbia pulcherrima –* Full protein sequence

MVVKVYGAAQAACPQRVMACLLEKDIPFDLVHVDLPSAQHKLSSFLLKQPFGLVPAIEDGDFRLFESRAIMRYYATKYEERGPNLLGKTLEEKAIVDQWVEVEAHNFNNLVYNIVIEVLIKPKMGEQGDINIVKSCEHKLDKVFDVYEERLSSSKYLGGDYFTLADLTHMPSIRYLVHELGLAHLVHNRNKVNAWWIDISDRPAWKNLMILAGY

>Bract1 – *Euphorbia pulcherrima –* Truncated protein sequence

MVVKVYGAAQAACPQRVMACLLEKDIPFDLVHVDLPSAQHKLSSFSNSPLG
