# Supplementary material for: A highly mutable GST is essential for bract colouration in Euphorbia pulcherrima Willd. Ex Klotsch
Source: BMC Genomics. 2021 Mar 23;22:208. doi: 10.1186/s12864-021-07527-z (PMC7988969; doi:10.1186/s12864-021-07527-z)
Supplement: Supplementary file 2 — Additional file 2. Full length gel images referent to Fig. 3a, Fig. 3b and Fig. 6a from this publication. [file 12864_2021_7527_MOESM2_ESM.docx]

**Additional File S2. Full length gel images referent to Figure 3a, Figure 3b and Figure 6a from this publication.**


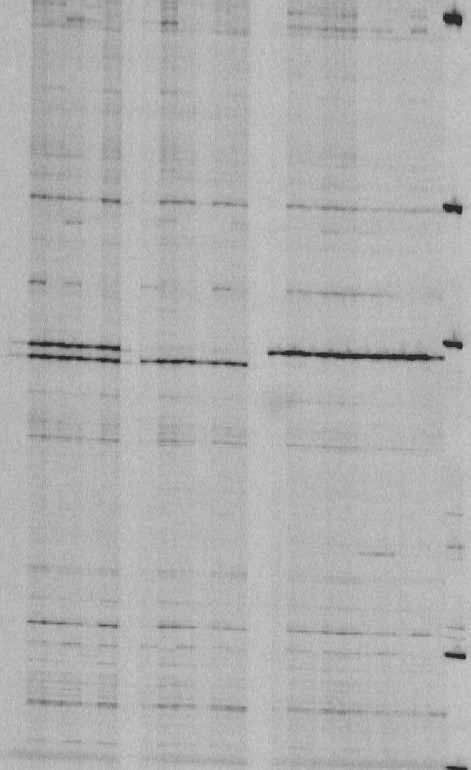


**Figure 1. Full length image referent to Figure 3a in this publication.** Band patterns from the amplified PCR fragments for *Bract1* in 22 red- and white-bracted poinsettia varieties. Sample order is the same as presented in Figure 3a from the publication. Last lane on the right side corresponds to DNA marker for size estimation. The contrast in the image displayed in the manuscript was adjusted to remove some of the grey background. The alteration does not alter the interpretation of the results.


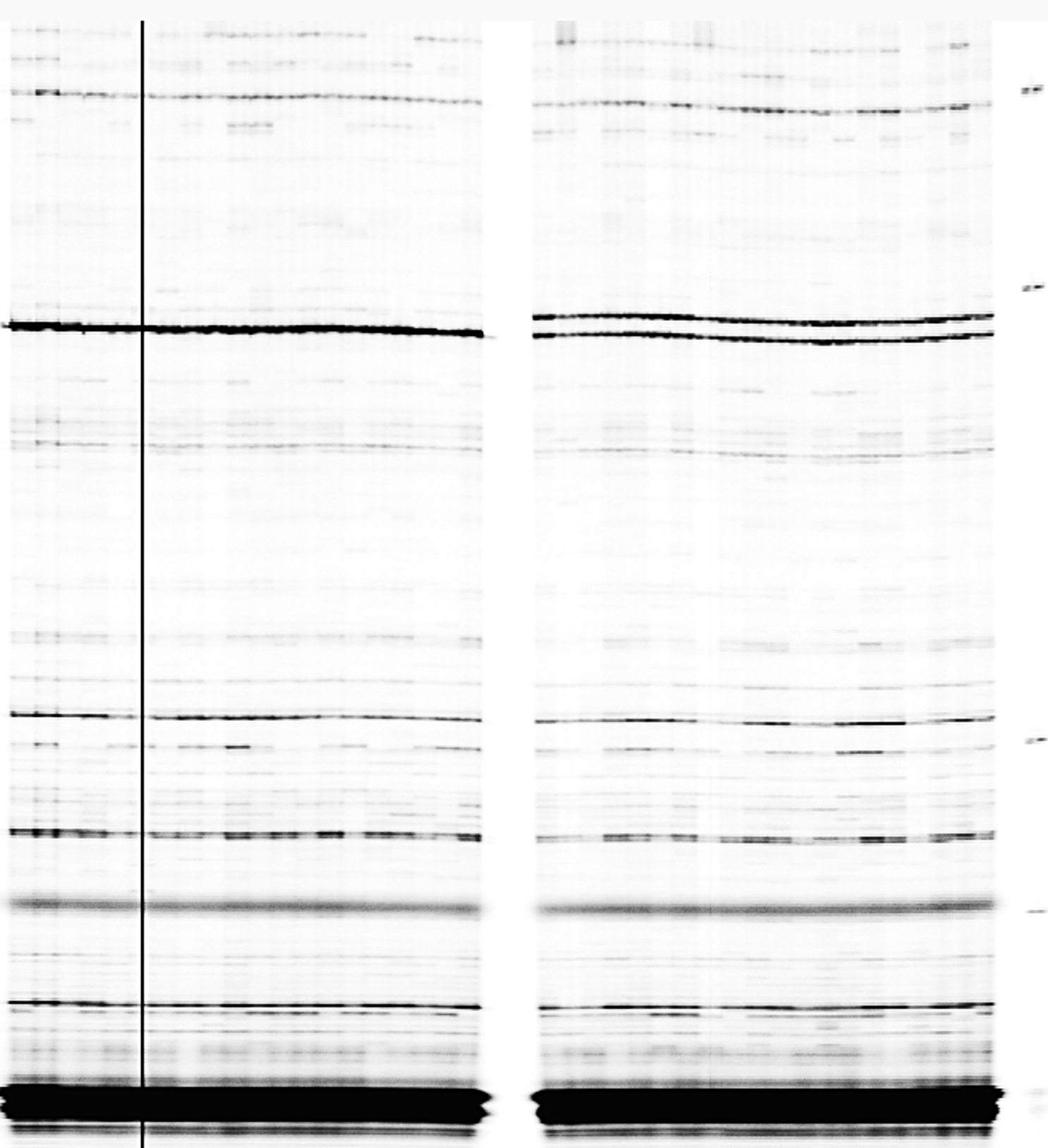


**Figure 2. Full length image referent to Figure 3b in this publication.** Example of the amplified PCR fragments for Bract1 for the segregating population ‘Joy’ (Rr) x ‘Joy White’ (rr). Sample order is the same as presented in Figure 3b from the publication. Last lane on the right side corresponds to DNA marker for size estimation. The black line seen in the left side of the image is due to a problem in the image capturing step. This was removed from the image displayed in the manuscript, but it does not alter the interpretation of the results. The samples cropped on the left side of the line also correspond to white (rr) progenies.


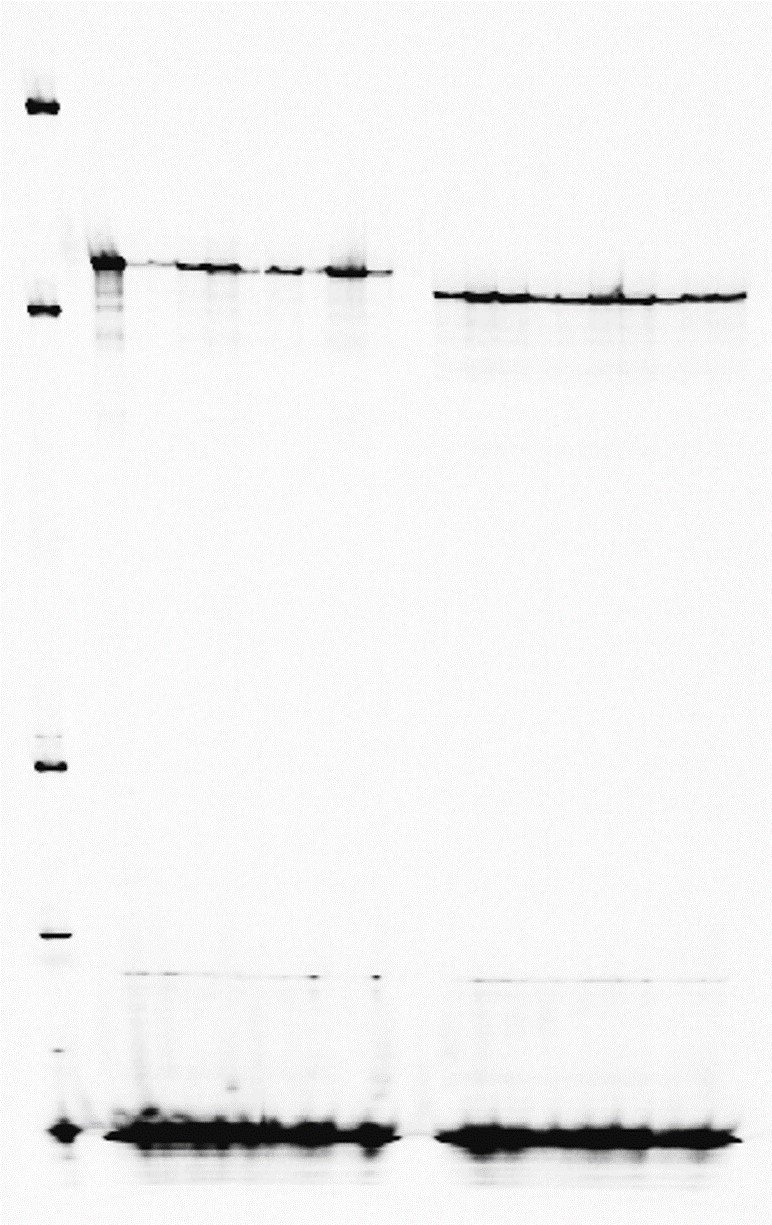


**Figure 3. Full length image referent to Figure 6a in this publication.** Genotyping of 10 independent biological replicates from the T2 progeny of *tt19/35S::Bract1* and *tt19/35S::Bract1_mut* transgenic plants. Sample order is the same as presented in Figure 6a from the publication. The image in the publication was cropped to remove part of the image that does not contain any information and to be able to create a composite figure in the publication. The alteration does not alter the interpretation of the results.
