# Supplementary material for: A highly mutable GST is essential for bract colouration in Euphorbia pulcherrima Willd. Ex Klotsch
Source: BMC Genomics. 2021 Mar 23;22:208. doi: 10.1186/s12864-021-07527-z (PMC7988969; doi:10.1186/s12864-021-07527-z)
Supplement: Supplementary file 3 — Additional file 3 Sequence similarity of Bract1 with other anthocyanin-related GSTs. [file 12864_2021_7527_MOESM3_ESM.docx]

**Additional File S3. Sequence similarity of *Bract1* with other anthocyanin-related GSTs.** Values on the upper and right parts of the table correspond to CDS nucleotide sequence similarities. Values on the lower and left parts of the table correspond to protein sequence similarities.

| **Genes** | **Bract1** | **AtTT19** | **PhAN9** | **CkmGST3** | **VvGST4** | **LcGST4** | **Riant1** | **Riant2** | **AtGSTF11** |  |
| --- | --- | --- | --- | --- | --- | --- | --- | --- | --- | --- |
| **Bract1** |  | 60,4% | 60,8% | 63,2% | 66,8% | 65,5% | 68,2% | 49,6% | 60,7% | **Nucleotide sequence similarity** |
| **AtTT19** | 55,1% |  | 52,2% | 58,9% | 62,4% | 62,7% | 62,1% | 50,5% | 75,3% |  |
| **PhAN9** | 53,8% | 46,1% |  | 63,6% | 61,9% | 60,3% | 61,8% | 46,7% | 53,3% |  |
| **CkmGST3** | 63,5% | 52,8% | 64,6% |  | 67,1% | 69,3% | 69,4% | 50,8% | 59,6% |  |
| **VvGST4** | 65,8% | 57,0% | 58,6% | 68,0% |  | 70,6% | 74,8% | 53,7% | 62,3% |  |
| **LcGST4** | 62,6% | 57,9% | 59,9% | 66,8% | 69,6% |  | 71,7% | 53,6% | 61,0% |  |
| **PpRiant1** | 66,5% | 56,2% | 59,9% | 70,6% | 75,8% | 72,5% |  | 67,8% | 61,5% |  |
| **PpRiant2** | 40,7% | 37,9% | 40,0% | 44,4% | 45,3% | 46,2% | 58,7% |  | 47,3% |  |
| **AtGSTF11** | 58,4% | 68,6% | 47,8% | 53,7% | 58,8% | 56,5% | 57,6% | 37,0% |  |  |
|  | **Protein sequence similarity** | | | | | | | | |  |
