# Supplementary material for: A highly mutable GST is essential for bract colouration in Euphorbia pulcherrima Willd. Ex Klotsch
Source: BMC Genomics. 2021 Mar 23;22:208. doi: 10.1186/s12864-021-07527-z (PMC7988969; doi:10.1186/s12864-021-07527-z)
Supplement: Supplementary file 5 — Additional file 5 Deducted protein sequences from the Bract1 and 95 GSTs from E. pulcherrima GSTs, as well as anthocyanin-related GSTs from other species. [file 12864_2021_7527_MOESM5_ESM.docx]

**Additional File S5. Deducted protein sequences from the *Bract1* and 95 GSTs from *E. pulcherrima* GSTs, as well as anthocyanin-related GSTs from other species.** Protein sequences were used to construct a phylogenetic tree with MEGA X v10.0.5 using the maximum likelihood (ML) method with the Whelan And Goldman matrix-based model using a discrete gamma distribution (WAG+G). The tree topology was tested via a bootstrap analysis with 1000 replicates.

>AtGSTF11 - *Arabidopsis thaliana* – GenBank - NM_111189.3

MVVKVYGQIKAANPQRVLLCFLEKDIEFEVIHVDLDKLEQKKPQHLLRQPFGQVPAIEDGYLKLFESRAIARYYATKYADQGTDLLGKTLEGRAIVDQWVEVENNYFYAVALPLVMNVVFKPKSGKPCDVALVEELKVKFDKVLDVYENRLATNRYLGGDEFTLADLSHMPGMRYIMNETSLSGLVTSRENLNRWWNEISARPAWKKLMELAAY

>AtTT19 - *Arabidopsis thaliana* - GenBank - NM_121728.4

MVVKLYGQVTAACPQRVLLCFLEKGIEFEIIHIDLDTFEQKKPEHLLRQPFGQVPAIEDGDFKLFESRAIARYYATKFADQGTNLLGKSLEHRAIVDQWADVETYYFNVLAQPLVINLIIKPRLGEKCDVVLVEDLKVKLGVVLDIYNNRLSSNRFLAGEEFTMADLTHMPAMGYLMSITDINQMVKARGSFNRWWEEISDRPSWKKLMVLAGH

>CkmGST3 - *Cyclamen persicum x Cyclamen purpurascens* - GenBank - AB682678.1

MVVKVYGPATAGCPQRVIACLFELDVDFEIIHVDLESGEHKKPDFLLRQPFGQVPAIEDGDFRLFESRAIMRYYAAKYSEKNPDLQGSTLEEKALVDQWLEVESHNFNDLVYTLVLHLMVFPQMGKRSDMQLVQECESKLEKVFDIYEERLSKSNYLAGKLFTLADLSHLPSITFLMGEGGLGHMVRNRKNVNSWWMDISSRPSWKKVRKLMD

>Bract1 – *Euphorbia pulcherrima*

MVVKVYGAAQAACPQRVMACLLEKDIPFDLVHVDLPSAQHKLSSFLLKQPFGLVPAIEDGDFRLFESRAIMRYYATKYEERGPNLLGKTLEEKAIVDQWVEVEAHNFNNLVYNIVIEVLIKPKMGEQGDINIVKSCEHKLDKVFDVYEERLSSSKYLGGDYFTLADLTHMPSIRYLVHELGLAHLVHNRNKVNAWWIDISDRPAWKNLMILAGY

>VvGST4 - *Vitis vinifera* - GenBank - AY971515.1

MVMKVYGPVRAACPQRVLACLVEKGVEFEVVHVDLDSGEQKRPDFLLRQPFGQVPVVEDGDFRLFESRAIVRYIAAKYAEQGPDLLGKSLEEKAVVDQWLEVEAHNFNELVYTLVMQLVILPRMGERGDLALAHTCEQKLEKVFDVYEQRLSKSRYLAGDSFTLADLSHLPAIRYLVKEAGMAHLVTERKSVSAWWEDISNRAAWKKVMELAA

>LcGST4 - *Litchi chinensis* - GenBank - KT946768.1

MVVKVYGPVTAGCPQRVMTCLLEKDVEFEIIHVNIDNGEHKRPEFLLRQPFGQVPVIEDDDFKLFESRAILRYYAAKYADRGPNLLGTTLEERARVDQWLEVEAHNFNDLIYTMVLQLIVIPSMGQPGDLTLVHSCEQKLEAVFDVYEKQLSKSKYLAGDWFSLADLSHMPALRFLMEDAKLVHLVKERKHVNAWWEEISGRLSWKKLMKLAYY

>PhAN9 - *Petunia hybrida* - GenBank - Y07721.1

MVVKVHGSAMAACPQRVMVCLIELGVDFELIHVDLDSLEQKKPEFLVLQPFGQVPVIEDGDFRLFESRAIIRYYAAKYEVKGSKLTGTTLEEKALVDQWLEVESNNYNDLVYNMVLQLLVFPKMGQTSDLTLVTKCANKLENVFDIYEQRLSKSKYLAGEFFSLADLSHLPSLRFLMNEGGFSHLVTKRKCLHEWYLDISSRDSWKKVLDLMMKKISEIEAVSIPAKEEAKV

>PpRiant1 - *Prunus persica* - GenBank - KT312847.1

MVVKVYGPVKAACPQRVMVCLLEKGVNFEIVDVNLEVGEQKQPQFLSRQPFGQVPAVEDGDFRLFESRAIIRYYAAKYADRGPNLLGTTLEEKALVDQWLEVEAHNFNDLVYTLVLQLLVLPRMGERGDVALVHACEEKLEKVFDVYEERLSKSSYLAGEAFTLADLSHLPGISYLIDEAKLGHLVSERKNVNAWWKDISNRPAWKKLMSLASDY

>PpRiant2 - *Prunus persica* - GenBank - KT312848.1

MVVKVYGPVKAACPQRVMVCLLEKGVNFEIVDVNLEVGEQKQPQFLSRQPFGQVPAVEDGDFRLFESRAIIRYYAAKYADRGPNLLGTTLEEKALVDQWLEVEAHNFNDLVYTLVLQLLVLPDRMGERGDVALVHACEEKLEKVFDVYEERLSKSSYLAGEAFTLADLSHLPGISYLIDEAKLGHLVSERKNVNAWWKDISNRPAWKKLMSLASDY

>T_DN34235_c0_g1_i2

MWKIYRTEGEEQEEAVKATSEMLQIIEEKAMGLVGDKKYFGGDNIGIVDIAYCVVAHWLGVMEKVAGIEILDPRKFPKLHAWTVNFKQAPVITENLPDSDEMVALFKRRRETILSSALKLQ

>T_DN39582_c0_g1_i1

MGEEVKLIGGWASPFSQRIELALKLKGVKYEYIEEDLFNKSPLLLKSNPIHKQVPVLIHNNKPISESPVILHYIDEAWPNSPLLPPNPYDKAIARFWAHFADTVCFPND

>T_DN40316_c0_g1_i1

MGEEVKVLGIWGSPFCCRIELALKLKGVEYQYIQQDLQNKSNILLNSNPVHQKVPVLVHNGKSIPESLVILEYIDHTWPQNPIFPRCPYARASARFWIKLVEEKLMQTVYKTTEASREEIEQLKQEFNGNLKLIENQLKDKDFFGGERIGCLDIVMIVLSHWFKVVREQVFNIEFISPDNFPVLHKWMTKVSQIDFVKESLPPPDKLTAFIRSRLHPPNSASV

>T_DN40316_c0_g1_i2

MQLMQTVYKTTEASREEIEQLKQEFNGNLKLIENQLKDKDFFGGERIGCLDIVMIVLSHWFKVVREQVFNIEFISPDNFPVLHKWMTKVSQIDFVKESLPPPDKLTAFIRSRLHPPNSASV

>T_DN43615_c2_g2_i1

MAVLGMWLEVEAHHFEPAASKLNWEIVFKPMFGMQADPAAVEENEEKLAKVLDVYESRLAQSKYLACDCFTLVDLHHLPNLQLLLGTQCKKLFDARPHVGAWAADITARPAWAKVLALQKQ

>T_DN43615_c2_g2_i2

MAAIKVHGTPISTATQRVLACLHEKGLDFEFNFVNLATGEHKQEPFISLNPFGKVPALEHGDLKLFESRAITRYITSENAEKGANLLCQGKQMAVLGMWLEVEAHHFEPAASKLNWEIVFKPMFGMQADPAAVEENEEKLAKVLDVYESRLAQSKYLACDCFTLVDLHHLPNLQLLLGTQCKKLFDARPHVGAWAADITARPAWAKVLALQKQ

>T_DN43615_c2_g2_i3

MAVLGMWLEVEAHHFEPAASKLNWEIVFKPMFGMQADPAAVEENEEKLAKVLDVYESRLAQSKYLACDCFTLVDLHHLPNLQLLLGTQCKKLFDARPHVGAWAADITARPAWAKVLALQKQ

>T_DN43615_c2_g4_i1

MAAIKVHGSPISTATQRVLACLHEKGLEYEFIHVNLATGEHKKQPFISLNPFGLVPAVEHGDLKLIESRAITQYITRENNEKGTNLLCEGKQMAVLGMWLEIEAHHFDPAASKLNWEVVLKPLFGHQTDPAAVEENEAKLGKVLDVYESRLAESKYLACDCFTLADLHHLPNLQLLLGSESKKLIDARPHVSAWAADITSRPAWAKVLALQKQ

>T_DN43622_c1_g1_i1

MLDIRWLISICNFKFFSGPFCQRVLLTIEEKHLPYDMKLVDLGNKPEWFLKLSPEGKVPVVKLEDKWVPDSDVITQTLEEKFPEPPLVTPPEKASVYVRLISY

>T_DN43622_c1_g1_i3

MLDIRWLISICNFKFFSGPFCQRVLLTIEEKHLPYDMKLVDLGNKPEWFLKLSPEGKVPVVKLEDKWVPDSDVITQTLEEKFPEPPLVTPPEKASVYVRLISY

>T_DN43622_c1_g1_i9

MLDIRWLISICNFKFFSGPFCQRVLLTIEEKHLPYDMKLVDLGNKPEWFLKLSPEGKVPVVKLEDKWVPDSDVITQTLEEKFPEPPLVTPPEKASVYVRLISY

>T_DN43892_c1_g1_i10

MAAAPVKVYGPAFSTAVSRVLACLVEKDVDFQLIPVNMSKGEHKMPDFLKIQPFGQVPAFQDESVSLFESRSICRYICEKHIDKGNKELYGTNPLAKASIDQWLEAEGQSFNPPSGALVFQLAFAPRMKIPQDEKLIRQNEEKLKKVLDIYEKRLGESRFLAGEEFSLADLSHLPNSQYLIAATDRGELFSSRKNVQRWWDEISSRESWKKVVQMQKSG

>T_DN43892_c1_g1_i11

MAAAPVKVYGPAFSTAVSRVLACLVEKDVDFQLIPVNMSKGEHKMPDFLKIQPFGQVPAFQDESVSLFESRSICRYICEKHIDKGNKELYGTNPLAKASIDQWLEAEGQSFNPPSGALVFQLAFAPRMKIPQDEKLIRQNEEKLKKVLDIYEKRLGESRFLAGEEFSLADLSHLPNSQYLIAATDRGELFSSRKNVQRWWDEISSRESWKKVVQMQKSG

>T_DN44709_c2_g1_i1

MALKLCVHRVSQPSRALLIFCKSNGIEFEEVVIDLSNKQHKSPEFLEINPMGQVPAMVHGDFKLFESHAILTYLAAAFPQVADDWYVLIYLAMAFPQVADHCPSDIKQRAKVQSVLDWHHSNLRRGSANYIFNSKLAPAFGLPLNPEAQLILSTQN

>T_DN44709_c2_g1_i10

MALKLYVHRTSQPSRSLIVFCKVNGIEFEEVTLDLTNKQHRSPEFLEINPLGQVPAMVHGDFKLFESHAILTYLAAAFPQVADDWYVLIYLAMAFPQVADHWYPSDIFQRAKVQSVLDWHHSNLRRGSANYIFNSKLAPAFGLPLNPEAQLILSTQN

>T_DN44709_c2_g1_i11

MGQVPAMVHGDFKLFESHAILTYLAAAFPQVADDWYVLIYLAMAFPQVADHCPSDIKQRAKVQSVLDWHHSNLRRGSANYIFNSKLAPAFGLPLNPEAQLILSTQN

>T_DN44709_c2_g1_i13

MGQVPAMVHGDFKLFESHAILTYLAAAFPQVADDWYVLIYLAMAFPQVADHCPSDIKQRAKVQSVLDWHHSNLRRGSANYIFNSKLAPAFGLPLNPEAQLILSTQN

>T_DN44709_c2_g1_i14

MALKLYVHRTSQPSRSLIVFCKVNGIEFEEVTLDLTNKQHRSPEFLEINPLGQVPAMVHGDFKLFESHAILTYLAAAFPQVADDWYVLIYLAMAFPQVADHWYPSDIFQRAKVQSVLDWHHSNLRRGSGDLFDIVKMHKLSLQFSDARIGINSFFQLCFCSIPDSL

>T_DN44709_c2_g1_i5

MGQVPAMVHGDFKLFESHAILTYLAAAFPQVADDWYVLIYLAMAFPQVADHCPSDIKQRAKVQSVLDWHHSNLRRGSANYIFNSKLAPAFGLPLNPEAQLILSTQN

>T_DN44709_c2_g1_i8

MSPYSITILLISLTEYLLSFFPGNFLFLNVLGSVTATYTFNAKLAPHFNIPLNPQAAAEGEKILYQSLSTIESFWLNDTGKFLVGGDKPSVADLSLACEVMQLEASNLLLAKFFTLFKVYESYEKEIVLSWINKFSIQLMEEDDRKRILGPFKKVQQWVENTKNAINPHFDEVHETVLQVRDMLMKKQPTGEKSEA

>T_DN44774_c11_g1_i1

MQFYHHPYSMDSQKVRIALEEKGIDYTSHHVNPITGKNMDSSFFRRNPTAKLPVFQNGNHIIFDTIEIIQYVSPLPHPLNLPPFSIFNFGKILCCRYIERIAVVSSGADETSFSSQEVIEWMRKIQQWNPKFFTLSHIPEKYRKTVSKFLRRVVIARMAEFPDLASAYHRKLKEAYETEDKLKNPEVLNRSKEHLLRVLDEVEAKLSETSYLAGEEFTMADAMLIPVLARLVLLNLEQEYISSRPNIAEYWVLVQQRPSYKKVIGKYFKGWRRYRTLVKTWCFVRIRSLLRKY

>T_DN44774_c11_g1_i2

MQFYHHPYSMDSQKVRIALEEKGIDYTSHHVNPITGKNMDSSFFRRNPTAKLPVFQNGNHIIFDTIEIIQYIERIAVVSSGADETSFSSQEVIEWMRKIQQWNPKFFTLSHIPEKYRKTVSKFLRRVVIARMAEFPDLASAYHRKLKEAYETEDKLKNPEVLNRSKEHLLRVLDEVEAKLSETSYLAGEEFTMADAMLIPVLARLVLLNLEQEYISSRPNIAEYWVLVQQRPSYKKVIGKYFKGWRRYRTLVKTWCFVRIRSLLRKY

>T_DN44774_c11_g1_i3

MQFYHHPYSMDSQKVRIALEEKGIDYTSHHVNPITGKNMDSSFFRRNPTAKLPVFQNGNHIIFDTIEIIQYIERIAVVSSGADETSFSSQEVIEWMRKIQQWNPKFFTLSHIPEKYRKTVSKFLRRVVIARMAEFPDLASAYHRKLKEAYETEDKLKNPEVLNRSKEHLLRVLDEVEAKLSETSYLAGEEFTMADAMLIPVLARLVLLNLEQEYISSRPNIAEYWVLVQQRPSYKKVIGKYFKGWRRYRTLVKTWCFVRIRSLLRKY

>T_DN45356_c1_g1_i2

MNTLNKIWLTRALCSLKTTQFTKRSLYLFTMANQLWRHLLFLNKLMKLGKAILSFLQIPMTEPWHDSGLSSLMKRYLYFLLHFEHFTFFLQFNQVNERNLDQLFI

>T_DN45356_c1_g1_i4

MGEVKVLGSCLSPFSRRVELALKLKGVEYEYIEQNLANKSSLLLENNPIHKKVPVLIHNGKPIVESFVILEYIDETWKGYPIFPAEPYDRAMARFWAKFIDEKCNDATWKIISSKGEEREKGIEEARQHLKTLENELKNKIFFGGEMIGAVDIAASFIGYWVLILQEAMGLDLVTKDAFPILCNWIHQYLSSEIVKQNLPPRDVVYDKFYALLHSPFNAKDWYY

>T_DN45356_c1_g1_i5

MGEVKVLGSCLSPFSRRVELALKLKGVEYEYIEQNLANKSSLLLENNPIHKKVPVLIHNGKPIVESFVILEYIDETWKGYPIFPAEPYDRAMARFWAKFIDEKCNDATWKIISSKGEEREKGIEEARQHLKTLENELKNKIFFGGEMIGAVDIAASFIGYWVLILQEAMGLDLVTKDAFPILCNWIHQYLSSEIVKQNLPPRDVVYDKFYALLHSPFNAKDWYY

>T_DN45465_c6_g4_i2

MPHQRTTADKAKKLEKMALKLYVHRASQPSRALIIFCKLNGIEFEEVFIDLTKKQHKSPEFLGINPMGQLPAMVHGDFKLFESHAILIYLASAFPKVADHWFPSDIIQRAKVQSVLDWHHSNLRRGSVTYTFNAKFARAFGLPLNPEAAAEGEKLLTASLSIIESFWLSESGKFLVGGDKPSIADLSLVCEIMQLEFMGEDNRKRILEPFMKVQEWIENTKLAMAPHFDEVHNSIFSQVRALEMIKEQKSTEEKSEA

>T_DN45963_c3_g1_i1

MEVDINVLRWFKPRMGYIPYLLPVEDTAIDATKRALGALNTYLASNTHLVGNSVTLADIVLICNLYLGFAYVMTKSFTLDFPNVERYFWKMVNEPNFKKIMGDIEQPESYPPVYTTPFLEQ

>T_DN45963_c3_g1_i2

MAMLLYAGVAGKLKNKNAWKVQIAAEYCGVELKVADNFEFGVTNKTSDFTKMNPIGKAPVLVTSDGPLFESNAISRYIARLKPDSPLYGSSLIDYARVEQWIDFASMEVDINVLRWFKPRMGYIPYLLPVEDTAIDATKRALGALNTYLASNTHLVGNSVTLADIVLICNLYLGFAYVMTKSFTLDFPNVERYFWKMVNEPNFKKIMGDIEQPESYPPVYTTPFLEQ

>T_DN45963_c3_g1_i4

MEVDINVLRWFKPRMGYIPYLLPVEDTAIDATKRALGALNTYLASNTHLVGNSVTLADIVLICNLYLGFAYVMTKSFTLDFPNVERYFWKMVNEPNFKKIMGDIEQPESYPPVYTTPFLEQ

>T_DN46449_c2_g1_i1

MGKDRNIGIAMDFSKGSKLALNWAINNLVDSGDTLYVIHVKPSQGNETRNLLWATTGSRNYPFCFGFKALLVMQARCLIFVLYEIFVALIPLVEFRAKDVAKEYEIHLDPEVLDMLDTVSRQKQVSFSSSNLLLIFWLFTVYSFSW

>T_DN46449_c2_g1_i2

MEEEVKLLGTWASPFSHRIKLALTLKQIQYEYIEQDLINKSTLLLKSNPVHKKIPVLIHNQKPISESLVILEYIDQIWLNIPFLPKQPHHRATARFWAKFVDEKILQTALKGSAAMGEEKEQIKEEIEEYLKLLENELKGNDYFGGENIGYVDIVAFSISYFFKIRQRIMRIELINEDKFPGLYNWMGKMCEIDAVNQSLPPSALHFANNVANQESP

>T_DN46449_c2_g1_i5

MEEEVKLLGTWASPFSHRIKLALTLKQIQYEYIEQDLINKSTLLLKSNPVHKKIPVLIHNQKPISESLVILEYIDQIWLNIPFLPKQPHHRATARFWAKFVDEKILQTALKGSAAMGEEKEQIKEEIEEYLKLLENELKGNDYFGGENIGYVDIVAFSISYFFKIRQRIMRIELINEDKFPGLYNWMGKMCEIDAVNQSLPPSALHFANNVANQESP

>T_DN46514_c3_g1_i1

MASNKDEATLLDFWASPYGMRLRIALAEKGIKYEYKEEDLRNKSPLLLQMNPVHKKIPVLIHNGKPIAESLIAVEYIDEVWADKAPLLPSHPYHRAQARFWADFADKKVFVLLNG

>T_DN46514_c3_g1_i2

MASNKDEATLLDFWASPYGMRLRIALAEKGIKYEYKEEDLRNKSPLLLQMNPVHKKIPVLIHNGKPIAESLIAVEYIDEVWADKAPLLPSHPYHRAQARFWADFADKKIYDLGRKLWATKGEEKEEAKKGFTECLKLLEAELGDKPYFGGQSLGYVDVAFVPFYAWFYAYEVEGNLNLEAEFPKLIQWGKRCMLKESVSKSVPDQKKIHGFVLELKKMFGIE

>T_DN46514_c3_g1_i3

MASNKYEVTLLDFWASPFGTRLRIALAEKGIKYEYKEEDLRNKSPLLLQMNPVHKKIPVLIHNGKPIAESLIAVEYIDEVWADKAPLLPSHPYHRAQARFWADFIDKKIYDLGRKLWATKGEEKEEAKKGFTECLKLLEAELGDKPYFGGQSLGYVDVAFVPFYAWFYAYEVEGNLNLEAEFPKLIQWGKRCMLKESVSKSVPDQKKIHGFVLELKKMFGIE

>T_DN46514_c3_g1_i4

MASNKDEATLLDFWASPYGMRLRIALAEKGIKYEYKEEDLRNKSPLLLQMNPVHKKIPVLIHNGKPIAESLIAVEYIDEVWADKAPLLPSHPYHRAQARFWADFADKKCCFLQEH

>T_DN46514_c3_g1_i5

MASNKDEATLLDFWASPYGMRLRIALAEKGIKYEYKEEDLRNKSPLLLQMNPVHKKIPVLIHNGKPIAESLIAVEYIDEVWADKAPLLPSHPYHRAQARFWADFADKKVFVLLNG

>T_DN46726_c0_g1_i1

MFNSNFNDIAENAALDLYPPHLRALIDETNEWVYSGINNGVYKCGFARKQEPYEEAMIQLYEALDKCENILEKQRYICGNALSEADIRLFVTLVRFDEVYAVHFKCNKKLLREYPNLFNYTKDIFQIPGMSSSVNMEHIKRHYYGSHPSINPFGIIPLGPDIDFSSPHDREKFGK

>T_DN46726_c0_g1_i10

MTEKSLAMIQLYEALDKCENILEKQRYICGNALSEADIRLFVTLVRFDEVYAVHFKCNKKLLREYPNLFNYTKDIFQIPGMSSSVNMEHIKRHYYGSHPSINPFGIIPLGPDIDFSSPHDREKFGK

>T_DN46726_c0_g1_i18

MARSALDEMSESGAFKRTASTFRDSISNEPDSPFPPESGRYHLYISYACPWASRCLAYLKIKGLEKAVTFSSVKPVWGRTKETDEHMGWVFPASDTEEPGAHPDILNGAKSIRDLYEIASSNYTGKYTVPVLWDKKLKTIVNNESSDIIRMFNSNFNDIAENAALDLYPPHLRALIDETNEWVYSGINNGVYKCGFARKQEPYEEAMIQLYEALDKCENILEKQRYICGNALSEADIRLFVTLVRFDEVYAVHFKCNKKLLREYPNLFNYTKDIFQIPGMSSSVNMEHIKRHYYGSHPSINPFGIIPLGPDIDFSSPHDREKFGK

>T_DN46726_c0_g1_i2

MQAMIQLYEALDKCENILEKQRYICGNALSEADIRLFVTLVRFDEVYAVHFKCNKKLLREYPNLFNYTKDIFQIPGMSSSVNMEHIKRHYYGSHPSINPFGIIPLGPDIDFSSPHDREKFGK

>T_DN46726_c0_g1_i3

MARSALDEMSESGAFKRTASTFRDSISNEPDSPFPPESGRYHLYISYACPWASRCLAYLKIKGLEKAVTFSSVKPVWGRTKETDEHMGWVFPASDTEEPGAHPDILNGAKSIRDLYEIASSNYTGKYTVPVLWDKKLKTIVNNESSDIIRMFNSNFNDIAENAALDLYPPHLRALIDETNEWVYSGINNGVYKCGFARKQEPYEEAMIQLYEALDKCENILEKQRYICGNALSEADIRLFVTLVRFDEVYAVHFKCNKKLLREYPNLFNYTKDIFQIPGMSSSVNMEHIKRHYYGSHPSINPFGIIPLGPDIDFSSPHDREKFGK

>T_DN46726_c0_g1_i4

MARSALDEMSESGAFKRTASTFRDSISNEPDSPFPPESGRYHLYISYACPWASRCLAYLKIKGLEKAVTFSSVKPVWGRTKETDEHMGWVFPASDTEEPGAHPDILNGAKSIRDLYEIASSNYTGKYTVPVLWDKKLKTIVNNESSDIIRMFNSNFNDIAENAALDLYPPHLRALIDETNEWVYSGINNGVYKCGFARKQEPYEEAMIQLYEALDKCENILEKQRYICGNALSEADIRLFVTLVRFDEVYAVHFKCNKKLLREYPNLFNYTKDIFQIPGMSSSVNMEHIKRHYYGSHPSINPFGIIPLGPDIDFSSPHDREKFGK

>T_DN46726_c0_g1_i9

MARSALDEMSESGAFKRTASTFRDSISNEPDSPFPPESGRYHLYISYACPWASRCLAYLKIKGLEKAVTFSSVKPVWGRTKETDEHMGWVFPASDTEEPGAHPDILNGAKSIRDLYEIASSNYTGKYTVPVLWDKKLKTIVNNESSDIIRMFNSNFNDIAENAALDLYPPHLRALIDETNEWVYSGINNGVYKCGFARKQEPYEEAMIQLYEALDKCENILEKQRYICGNALSEADIRLFVTLVRFDEVYAVHFKCNKKLLREYPNLFNYTKDIFQIPGMSSSVNMEHIKRHYYGSHPSINPFGIIPLGPDIDFSSPHDREKFGK

>T_DN46759_c2_g1_i4

MVPSIGRFSFKLSRAKQKKKGMGEVKVLGAWASPYSYKVIWALKLKGIPFEYLEQDLVNKSELLLQCNPVYKKIPVLVHGGNPICESMIIVEYLDQTCPQYPLLPVDPYERALSRFWVKYIDDKITSMWMIYRSSGEEQEKAVKGTMEMLETIEEEAMRIVGDNKYFGGDKIGIVDIAFGEIAHWMGVIEKIVGIQVHVPSKFPKLHSWIQNFKQAPIICENLPDPEKLFAVFKPLRETILASTSVA

>T_DN46759_c2_g1_i6

MVPSIGRFSFKLSRAKQKKKGMGEVKVLGAWASPYSYKVIWALKLKGIPFEYLEQDLVNKSELLLQCNPVYKKIPVLVHGGNPICESMIIVEYLDQTCPQYPLLPVDPYERALSRFWVKYIDDKITSMWMIYRSSGEEQEKAVKGTMEMLETIEEEAMRIVGDNKYFGGDKIGIVDIAFGEIAHWMGVIEKIVGIQVHVPSKFPKLHSWIQNFKQAPIICENLPDPEKLFAVFKPLRETILASTSVA

>T_DN46773_c2_g1_i2

MMKLKVYADRMSQPSRAVIIFCKVNGIDFEEVTINLAKRQQLTPEFKEINPLGKVPAVVDGRFKLFESHAILIYLASVFPGVADHWYPADLFKRAKIQSVLDWHHTNLRRGAAGYVLNAKLAPALGLPLNPQAAAEAEKVLSSSLSKIESFWLKGKGRFLLGGSQPSIADLSLVCEIMQLEVLDEEDRNRFLGPHKKVQEWIEDTKNATRPHFDEVHKLLFKAKARLQKLNSETQTSSTTTLHSKM

>T_DN47630_c1_g3_i2

MPSKCLMFCFYESAYNINYVAIVFVCMFRLYISYTCPYAQRVWITRNCKGLQDQIELVPIDLQDRPAWYKEKVYPPNKVPALEHKNEVRGESLDLIKYIDSNFEGPSLFPDDPAKKELAEELFSHLDSFNTALRSLFKGDANEAGSALDFIETSLSKLTAGPFFLAQFSLVDIAYAPFVERFLPALLEVKEYDITSGRPNLAAWIQVFVYFCLGYG

>T_DN47630_c1_g3_i6

MEQLPVNWLYISYTCPYAQRVWITRNCKGLQDQIELVPIDLQDRPAWYKEKVYPPNKVPALEHKNEVRGESLDLIKYIDSNFEGPSLFPDVTHSLFLVFISLINE

>T_DN49420_c1_g1_i1

MDFSAKKKEKRVARMAVKLIGAWASPFVMRPRIALNIKSVDYEFLEETFGSKSQLLLESNPVHKKIPVLIHDGKPICESLVIVQYIDEVWSSAPSILPSDPYERAIARFWAAYVDDKFFPSLRSIRFAVGEAKEEAIKQVKEGLLLLEEALVKCGKGKPFFGGDEIGYLDIAFGSYLGWLRVTEVSIESQLITEANTPNLFNWATTFSSHPAVKDVLPETQKLVEFAKVIAAKMRPAA

>T_DN49420_c1_g1_i2

MAKSEVKLIGAWPSPFVMRPRIALNIKSVDYEFLEEAFGAKSQLLLESNPVYKKIPVLIHEGPPICESLVIVQYIDEVWSSAPSILPSDPYERAIARFWGSYVDDKFFPSLRGIIFAEGEARQEAIEQVKEMLVLLEEAFVKCSKGNPFFGGDEIGYLDIAFGSYLGWLRANEVSGETQLITEANTPNLFNWATAFSSHPAVKDVLPETHKLVEFGKLVAAKMRPAA

>T_DN49420_c1_g1_i4

MAKSEVKLIGAWPSPFVMRPRIALNIKSVDYEFLEEAFGAKSQLLLESNPVYKKIPVLIHEGPPICESLVIVQYIDEVWSSAPSILPSDPYERAIARFWGSYVDDKVSSLSCFLSLVSIFSN

>T_DN49599_c2_g3_i1

MLRLEYICMLQIYELGRKIWTTKGEELETAKEFIEALKLLEGELGDKAYYGGEDIGYMDVVFVPFSSWFYAFETCGNFSIEAECPKLIAWAKRCLQRDSISKSLPDSHKKDQSGGSKEYLQSTGA

>T_DN49599_c2_g3_i2

MANEVTLLDFWPSPFGMRLRIALAEKGIDYESREEDLRNKSPLLLQMNPVHKKIPVLIHNGKPIPESLIALQYIDEVWNHKSPLLPSDPYQRAQAGFWADFVDKKIYELGRKIWTTKGEELETAKEFIEALKLLEGELGDKAYYGGEDIGYMDVVFVPFSSWFYAFETCGNFSIEAECPKLIAWAKRCLQRDSISKSLPDSHKVYEFILMLKKKFGIE

>T_DN49638_c2_g1_i1

MIIIQYIDEVWNHKAPLLPSDPYQRAQARFWADYIDKKIYPNGSMLWATEGEVKETYKKNLIESFQTLERELGDKPYFGGEVFGYVDVALIPFYSMFYSFEKFGNFSMEVECPKIVEWATRCLQKETVSKSLSDPVKVYELHMWLTDVGLIKKLT

>T_DN49638_c2_g1_i2

MIIIQYIDEVWNHKAPLLPSDPYQRAQARFWADYIDKKIYPNGSMLWATEGEVKETYKKNLIESFQTLERELGDKPYFGGKIFGYVDVALIPFYSMFYSFEKFGNFSMEVECPKIVEWATRCLEKETVSKSIPDPIKVYELYMWLTDVGLIKKIGRAHV

>T_DN49638_c2_g1_i3

MLWATEGEVKETYKKNLIESFQTLERELGDKPYFGGKIFGYVDVALIPFYSMFYSFEKFGNFSMEVECPKIVEWATRCLEKETVSKSIPDPIKVYELYMWLTDVGLIKKIGRAHV

>T_DN49638_c2_g1_i7

MADEVTLVTFKISCYSERVKVALAEKGIKYEVKEENYETKSPLLLQLNPIHKQVPVLIHNGNPICESMIIIQYIDEVWNHKAPLLPSDPYQRAQARFWADYIDKKIYPNGSMLWATEGEVKETYKKNLIESFQTLERELGDKPYFGGKIFGYVDVALIPFYSMFYSFEKFGNFSMEVECPKIVEWATRCLEKETVSKSIPDPIKVYELYMWLTDVGLIKKLT

>T_DN49638_c2_g1_i8

MIIIQYIDEVWNHKAPLLPSDPYQRAQARFWADYIDKKIYPNGSMLWATEGEVKETYKKNLIESFQTLERELGDKPYFGGKIFGYVDVALIPFYSMFYSFEKFGNFSMEVECPKIVEWATRCLEKETVSKSIPDPIKVYELYMWLTDVGLIKKIGRAHV

>T_DN50435_c2_g1_i10

MNRERLMKMAGAVRTGGKGSMRRKKKAVHKTTTTDDKRLQSTLKRIGVNAIPAIEEVNIFKDDVVIQFVNPKVQASIAANTWVVSGTPQNKKLQDILPHVLSQLGPDNLDNLKKLAEQIQRQAPNAGAAAAPAEEDDDEVPELVAGETFEGAAAEEGKAAAS

>T_DN50435_c2_g1_i12

MLLSFVQMNRERLMKMAGAVRTGGKGSMRRKKKAVHKTTTTDDKRLQSTLKRIGVNAIPAIEEVNIFKDDVVIQFVNPKVQASIAANTWVVSGTPQNKKLQDILPHVLSQLGPDNLDNLKKLAEQIQRQAPNAGAAAAPAEEDDDEVPELVAGETFEGAAAEEGKAAAS

>T_DN50435_c2_g1_i13

MDQFFQMGEVKLIGTSASLFSRRIEWALKLKGVDYEFLQEDLMNKSPVLLKHNPVHKKVPVLVDGDKPVAESLVILEYIDDKWSHNPLLPSDPYQRAMARFWARFADDKCVMESFNAAWKEGEEKEKAIERAIESLGIIEEQIKGKKYFNGEKIGYSDLVMGWIPLWLSVMEQVGDMKLIDSNRFPSLYEWTHNFSEIPVIKDCLPPRDMLLNYFTASVAYMRSNSADLDKH

>T_DN50744_c4_g1_i1

MTKIVQMHGTGKNIMALEGEERAQAGQEMINALKMLEGELGDKAYFGGQSIGYVDVALLPFYQWFYTYEKLGIFNVEVSCPKLSEWGQRCLHKEHISKSIPPQEKLYSYFLDFNKES

>T_DN50744_c4_g1_i2

MAEEVILLNSLASPFGMRVSIALAEKGIKHEFRLEDLRNKSPLLLEMNPVHKMVPVLIHNGKPIVESHVIVQYIDEVWPGNGPLLPSHPYHRSQARFWADFVDKKMHGTGKNIMALEGEERAQAGQEMINALKMLEGELGDKAYFGGQSIGYVDVALLPFYQWFYTYEKLGIFNVEVSCPKLSEWGQRCLHKEHISKSIPPQEKLYSYFLDFNKES

>T_DN50744_c4_g1_i3

MTKIVQIHGTGKNIMALEGEERAQAGQEMINALKMLEGELGDKAYFGGQSIGYVDVALLPFYQWFYTYEKLGIFNVEVSCPKLSEWGQRCLHKEHISKSIPPQEKLYSYFLDFNKES

>T_DN50744_c4_g1_i4

MAEEVILLNSLASPFGMRVSIALAEKGIKHEFRLEDLRNKSPLLLEMNPVHKMVPVLIHNGKPIVESHVIVQYIDEVWPGNGPLLPSHPYHRSQARFWADFVDKKVPFFTPFVFFSFN

>T_DN51238_c5_g1_i3

MEESDGREGYTLVVRKPYFGLPTACPICLPVYIYLKLARFPFHLDFNSIYPDSDQIPYVESGVYVAFNNESGGVIQRLKDDAIVNLDVEFSSVPEWVSMESMISSWLIDAITYELWLGTDGSSAFKIYYSDLPWIIGKALFMKQVYTVKQQLGITKENVVRREEEIYKRAKIAYGALSTILGDQDFLFEDRPSSLDAFFLAHVLFTIQALPESSVIRTSVSEHGNLLRYAERLKSEFLEAASSSSEPSSSTARRAPSNSSSKAKRKPKKEKTEEEKTFKRRSKYFLVTQLVAVLLFLSVIGGYDFSEVDAGDEDEGYGYD

>T_DN51691_c0_g1_i14

MATLDKSVVENLPPPFDATADQPPLFDGTIRLYTCYTCPFAQRVWITRNYKGLQDTIKLVPLILQDRPAWYGEKVNPANKVPALEHNGKIIGESLDLTKYVDINFEGPSLLPDVRLAKLSKKSYLISSAYEV

>T_DN51691_c0_g1_i18

MYAAYRCPYAQRVWITRNYKGLQHTIKLVPLNLLNRPAWYGEKVYPVNKVPALEHNGKIIGESLDLIKYVDSNFEGPSLLPDDPAKKEFAEELFASTDKFLGTVFGSLKEADPVKLAGQFLEFSVVV

>T_DN51691_c0_g1_i4

MAALDKSVLENLPPPLDVTADQPPLFDGTIRMYAAYRCPYAQRVWITRNYKGLQHTIKLVPLNLLNRPAWYGEKVYPVNKVPALEHNGKIIGESLDLIKYVDSNFEGPSLLPDVRLAKLSIKAT

>T_DN51691_c0_g1_i5

MAALDKSVLENLPPPLDVTADQPPLFDGTIRMYAAYRCPYAQRVWITRNYKGLQDTIKLVPLDLLNRPAWYGEKVYPANKVPALEHNGKIIGESLDLIKYVDSNFEGPSLLPDDPAKKEFAEELFASTDKFLGTVFGSLKEADPVKLAGPKFDCLENILRKFDDGPFFLGSLFSLADIAYIPFVERYQIFFSDVYNYDITSGRPKLAVWIEEINKIQAYKQTKTDPKEIVAALKKIFQGQ

>T_DN51691_c0_g1_i7

MAALDKSVLENLPPPLDVTADQPPLFDGTIRMYAAYRCPYAQRVWITRNYKGLQHTIKLVPLNLLNRPAWYGEKVYPVNKVPALEHNGKIIGESLDLIKYVDSNFEGPSLLPDDPAKKEFAEELFASTDKFLGTVFGSLKEADPVKLAGQFLEFSVVV

>T_DN52213_c5_g2_i2

MYDPEGYSLWFCDYKYNDENTVSFVTLNKVGGFLQRMDLARKYAFGKMLVIGSNPPFKVKGLWLFRGPEIPKFVMDECYDMELYEWTKVDISDEAQKERVNQMIEDFEPFEGEPLLDAKCFK

>T_DN52213_c5_g2_i4

MGFTRIFTKSFTSEFPHVERYFWTMVNQPNFKKIIGEIKQAEAVPPVPKKPAHGKEPAKPKDAPKKEAKKEKEPPKPKEAVEEEEEAPKPKAKNPLDLLPPSKMILDDWKKLYSNTKTNFREVAIKGFWDMYDPEGYSLWFCDYKYNDENTVSFVTLNKVGGFLQRMDLARKYAFGKMLVIGSNPPFKVKGLWLFRGPEIPKFVMDECYDMELYEWTKVDISDEAQKERVNQMIEDFEPFEGEPLLDAKCFK

>T_DN52213_c5_g2_i5

MGFTRIFTKSFTSEFPHVERYFWTMVNQPNFKKIIGEVKQAESVPPVPKKPAHGKEPAKPKDAPKKEAKKEKEPPKPKEAVEEEEEAPKPKAKNPLDLLPPSKMILDDWKKLYSNTKTNFREVAIKGFWDMYDPEGYSLWFCDYKYNDENTVSFVTLNKVGGFLQRMDLARKYAFGKMLVIGSNPPFKVKGLWLFRGPEIPKFVMDECYDMELYEWTKVDISDEAQKERVNQMIEDFEPFEGEPLLDAKCFK

>T_DN52213_c5_g5_i1

MMPKSFTSEFPHVERHFWTMVNQPNFQKVIGDFKQAESVPPVAKKPAQAKEPAKPKDEPKKEAKKEKESPKPKEAAGEEEEAAPKPKAKNPLDLLPPSTMILDEWKRLYSNTKTNFRNVAIKGFWDMYDPEGYSLWFCDYKYNDENTVSFVTLNKVGGFLQRMDLARKYAFGKMLVIGSSPPFKVKGLWLFRGQEIPKFVMDECYDMELYEWTKVDITDEAQKERASQMIEDFEPFEGEPLLDAKCFK

>T_DN52213_c5_g5_i2

MYDPEGYSLWFCDYKYNDENTVSFVTLNKVGGFLQRMDLARKYAFGKMLVIGSSPPFKVKGLWLFRGQEIPKFVMDECYDMELYEWTKVDITDEAQKERASQMIEDFEPFEGEPLLDAKCFK

>T_DN52213_c5_g5_i4

MALVLYSGNYKNKNAYKALIAAEYSGVEIKLAENFEMGVTNKTPEFIKMNPIGKVPVLETPDGAVFESNAIARYVARQKADNPLYGSSLIDYAHIEQWIDFASLEIDANLLAWLKPRMGYAPYLPPVEEAVVAALKRGLGALNTHLATNTYLVGHSVTLADIILTCNLYLGFSLMMPKSFTSEFPHVERHFWTMVNQPNFQKVIGDFKQAESVPPVAKKPAQAKEPAKPKDEPKKEAKKEKESPKPKEAAGEEEEAAPKPKAKNPLDLLPPSTMILDEWKRLYSNTKTNFRNVAIKGFWDMYDPEGYSLWFCDYKYNDENTVSFVTLNKVGGFLQRMDLARKYAFGKMLVIGSSPPFKVKGLWLFRGQEIPKFVMDECYDMELYEWTKVDITDEAQKERASQMIEDFEPFEGEPLLDAKCFK

>T_DN52213_c5_g5_i5

MMPKSFTSEFPHVERHFWTMVNQPNFQKVIGDFKQAESVPPVAKKPAQAKEPAKPKDEPKKEAKKEKESPKPKEAAGEEEEAAPKPKAKNPLDLLPPSTMILDEWKRLYSNTKTNFRNVAIKGFWDMYDPEGYSLWFCDYKYNDENTVSFVTLNKVGGFLQRMDLARKYAFGKMLVIGSSPPFKVKGLWLFRGQEIPKFVMDECYDMELYEWTKVDITDEAQKERASQMIEDFEPFEGEPLLDAKCFK

>T_DN52222_c3_g1_i1

MSTIPIFSPPKYFWSPTSSIAFSSVVCNISNIFFTASSFSSPLVRYIFHIDVCLSSKNLTQNLAIALSEGSAGIKGYCCGHVSSKYSIIIIDSQIGFSPWMSTGIFL

>T_DN52222_c3_g1_i2

MGGVKLLGNWVSPYSYRVIWGLKLKGIPFEYVEQDFVTKSPLLLQSNPVHKKIPVLIHGENPICESMIIIEYLDETWPQQYPLMPADPSERAIARFWVKFFEDKVRLL

>T_DN52222_c4_g1_i1

MGEVVKLLGAWPSPYCYRVIWALKLKGIPFEYVEENLFDKSCLVVQHNPVYKKVPILVHAGNPINESMIIIEYLDQIWPQYPLLPTHPYHRALARFWVKYVEDKVLFDLLLPLRFL

>T_DN52222_c4_g1_i2

MGEVVKLLGAWPSPYCYRVIWALKLKGIPFEYVEENLFDKSCLVVQHNPVYKKVPILVHAGNPINESMIIIEYLDQIWPQYPLLPTHPYHRALARFWVKYVEDKDPYITTWKIFQTSGEEQEKAIQVNLEMLKSIEEEVMCLGLGDNKFFGGQNIGIVDIALGGLTHWLQAIETTLGIQVFDPLKFPKLHTWSQNFRQSPIISENLPNLDDMVALYIPRRKIVLASPSRKLY

>T_DN52827_c0_g3_i1

MSGSKDEVKLLGGWYSPFAFRVGLALKLKGIPYESVDEDLSNKSRLLLQLNPVHKKIPVLVHNGKPISESLVILEYIDDIWNHNPILPQHPYDRAMARFWANFIEEKFTGAVRGVLVAVDEEEKQKALEKAVEALGILEEELKKQGDSKFFGGDRVGLVDLTLSFFNNWVVAVEKAASVKIHNSEMFPLIEKWNQNFVELPFVKETLPQQDKLHDYFSKF

>T_DN52827_c0_g3_i2

MSGSKDEVKLLGGWYSPFAFRVGLALKLKGIPYESVDEDLSNKSRLLLQLNPVHKKIPVLVHNGKPISESLVILEYIDDIWNHNPILPQHPYDRAMARFWANFIEEKVYIQ

>T_DN52827_c0_g5_i2

MSGSQDEVKLIAGWFSLNVFRVELALKLKGIPYESLEEDFPKKSSLLLELNPVHNQIPVLVHNGKPICESLLILEYIDEIWEHNPILPQHPYDKAMARFWAKFVEEKVCIFIFFT

>T_DN52827_c0_g8_i1

MSIGSKDEVKVIGGWYSLGPFRVELALKLKGIPYEYLDEDLTNKSPLLLQLNPVYKQIPVLVHNGKPISESLVILEYIDEIWENNPILPQHPYDKAMARFWANFIDEKFTAAMKRILITFDEEEKQKEVENAIEALKILEEELEKKGNSKFFGGDTIGIADLTLSFLNNWLKGVENHTGTETQTETK

>T_DN52887_c3_g2_i1

MALEICVKAATGAPDVLGDCPFCQRVTLTLGEKKIPYKLHLVNLSDKPQWFLDISPEGKVPVVKFDDKWVPDSDMIVGILEEKYPEPSLVTPPEFGSAGSKIFPTFFKFVTNKDPNDGSEQALLEELKALDEHLKAHGPFIAGEKITAADLSLAPKLYHL

>T_DN53261_c1_g1

MCVEFLKLNPIGYVPVLVDGDVIISDSFAILMYLEEKYPQPPLLPHDLHKKAINYQAANIVSSSIQPFQNLAVLKVIEEKVSPDEKVPWAQFHISKGFAALEKLLQSHAGRFATGDEVYMADLFLQPQLHAAVTRFSVDMSKFPLLVRLHEEYNQLPAFQNATPDKQPDAPSSTTT

>T_DN53261_c1_g1_i10

MCVEFLKLNPIGYVPVLVDGDVIISDSFAILMYLEEKYPQPPLLPHDLHKKAINYQAANIVSSSIQPFQNLAVLKVIEEKVSPDEKVPWAQFHISKGFAALEKLLQSHAGRFATGDEVYMADLFLQPQLHAAVTRFSVDMSKFPLLVRLHEEYNQLPAFQNATPDKQPDAPSSTTT

>T_DN53261_c1_g1_i3

MCVEFLKLNPIGYVPVLVDGDVIISDSFAILMYLEEKYPQPPLLPHDLHKKAINYQAANIVSSSIQPFQNLAVLKVIEEKVSPDEKVPWAQFHISKGFAALEKLLQSHAGRFATGDEVYMADLFLQPQLHAAVTRFSVDMSKFPLLVRLHEEYNQLPAFQNATPDKQPDAPSSTTT

>T_DN53261_c1_g1_i5

MCVEFLKLNPIGYVPVLVDGDVIVSDSFAILMVSFTLSHSFSRFSKSLTDIYQQYLEEKYPQPPLLPHDLHKKAINYQVLSSTSLSGLLIAQYSPSFYP

>T_DN53261_c1_g2_i3

MKFMVDKVSTDEKFAWVQSHISRGFAALENLLQGHAGQFATGDEVFMADVFLQPQLHAAVTRFNIDMTKFPLLLRLHEAYNQLAAFQDAMPEKQPDAPSSTTT

>T_DN54173_c0_g1_i1

MLHLYTESRAITAYVCEKYKDTGYDLTRHKDLKEAAMVKVWAEVESQQYNPAITPIIHQHFVAPLKGESPDQSILKTNLEKLAKVLDIYETRLTNSKYLAGDFYSMADLHHLPYTYYLMKTEAASVINERPHVKAWWLDISSRPAFLKVAPGMTFGQN
