# Supplementary material for: A highly mutable GST is essential for bract colouration in Euphorbia pulcherrima Willd. Ex Klotsch
Source: BMC Genomics. 2021 Mar 23;22:208. doi: 10.1186/s12864-021-07527-z (PMC7988969; doi:10.1186/s12864-021-07527-z)

**Additional File S6. Schematic representation from the C757pGFPU10-35s-ocs-LH binary vector used for the constructions of transformation plasmids containing either the wild-type allele (*35S::Bract1*) or the mutated allele (*35S::Bract1_mut*) from the poinsettia GST.**


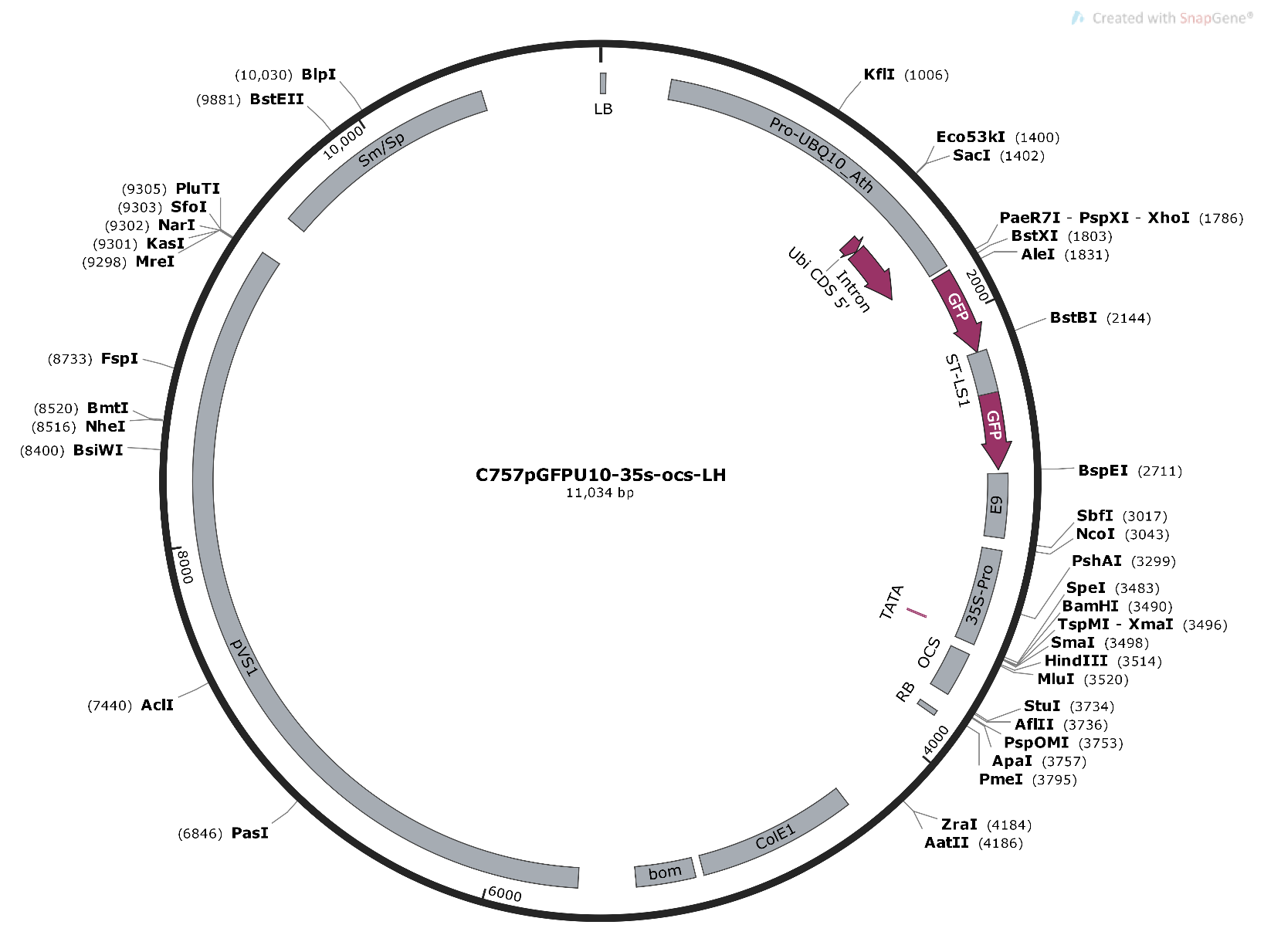

Supplement: Supplementary file 6 — Additional file 6 Schematic representation from the C757pGFPU10–35 s-ocs-LH binary vector used for the constructions of transformation plasmids containing either the wild-type allele (35S::Bract1) or the mutated allele (35S::Bract1_mut) from the poinsettia GST. [file 12864_2021_7527_MOESM6_ESM.docx]
