# Supplementary material for: A highly mutable GST is essential for bract colouration in Euphorbia pulcherrima Willd. Ex Klotsch
Source: BMC Genomics. 2021 Mar 23;22:208. doi: 10.1186/s12864-021-07527-z (PMC7988969; doi:10.1186/s12864-021-07527-z)
Supplement: Supplementary file 7 — Additional file 7 Sequence alignment of Bract1, anthocyanin-related GSTs and orthologue GSTs from Euphorbiaceae species. [file 12864_2021_7527_MOESM7_ESM.docx]

**Additional File S7.** **Sequence alignment of *Bract1*, anthocyanin-related GSTs and orthologue GSTs from Euphorbiaceae species.** The numbers in the alignments indicate the nucleotide positions in the CDS. Sequences were aligned using the ClustalW function on the BioEdit Sequence Alignment Editor v7.2.5.

....|....| ....|....| ....|....| ....|....| ....|....| ....|....| ....|....| ....|....|

5 15 25 35 45 55 65 75

*Bract1* ATGGTAGTGA AAGTGTATGG AGCAGCTCAG GCAGCTTGCC CACAAAGAGT AATGGCCTGC CTTTTAGAGA AAGATATTCC

*Euphorbia esula* ATGGTAGTTA AAGTTTATGG AGCAGTCCAA GCAGCTTGCC CACAGAGGGT ATTAGCTTGC CTATTGGAAA AAGATGTTCA

*Euphorbia pekinensis* ATGGTAGTGA AAGTGTATGG ACCATTGTAT GCAGCTTGCC CACAGAGGGT ATTAGCCTGC CTATTGGAGA AAGATGTTCA

*Ricinus communis* ATGGTAGTTA AAGTGTATGG TTCAGTGCAT GCTGCTTGCC CACAAAGGGT ATTGGCTTGC CTTTTGGAGA AAGATGTTGA

*Jatropha curcas* ATGGCAATTA AAGTTTATGG CCCTGCAAAG GCTGCTTGCC TACAAAGGGT ATTAGCTTGC TTTTTGGAAA AAGACCTCCA

*Hevea brasiliensis* ATGGTAGTGA AGGTTTATGG TCCAGTGCAA GCTGCCTGCC CACAGAGGGT ATTGGCTTGC CTTTTGGAGA AGGATGTGGA

*Manihot esculenta* ATGGTAGTAA AGGTTTATGG TCCAGTGCGA GCTGCCTGTC CACAGAGGGT TCTGGCTTGC CTTTTGGAGA AGGATGTGGA

*VvGST4* ATGGTGATGA AGGTGTATGG CCCAGTGAGG GCTGCATGCC CACAGAGGGT GTTGGCTTGC CTTGTAGAGA AGGGCGTGGA

*PpRiant1* ATGGTTGTGA AAGTGTATGG TCCAGTCAAG GCAGCCTGCC CTCAGAGGGT TATGGTTTGT CTTTTGGAGA AAGGAGTGAA

*PpRiant2* ATGGTTGTGA AAGTGTATGG TCCAGTCAAG GCAGCCTGCC CTCAGAGGGT TATGGTTTGT CTTTTGGAGA AAGGAGTGAA

*CkmGST3* ATGGTTGTTA AAGTGTATGG TCCAGCCACT GCAGGTTGCC CACAGAGGGT GATCGCTTGC CTTTTTGAAC TTGACGTCGA

*PhAN9* ATGGTTGTGA AAGTGCATGG TTCAGCAATG GCTGCATGCC CACAGAGGGT CATGGTCTGC CTGATAGAGT TGGGGGTTGA

*AtTT19* ATGGTTGTGA AACTATATGG ACAGGTAACA GCAGCTTGTC CACAAAGAGT CTTGCTTTGT TTTCTCGAGA AAGGAATTGA

*LcGST4* ATGGTGGTTA AAGTTTATGG ACCAGTTACG GCAGGTTGCC CACAGAGAGT TATGACTTGC CTTCTTGAAA AAGATGTTGA

....|....| ....|....| ....|....| ....|....| ....|....| ....|....| ....|....| ....|....|

85 95 105 115 125 135 145 155

*Bract1* TTTTGATCTT GTTCATGTTG ATCTTCCTTC TGCTCAACAT AAACTCTCTT CCTTCCTTCT CAAACAGCCC TTTGGGTTAG

*Euphorbia esula* ATTTGAAATT GTCCACGTGG ATCTTCATTC TGCTCAACAT AAACTCCCTT CTTACCTTTG CAAACAGCCC TTTGGGTTAG

*Euphorbia pekinensis* ATTTGAAATT GTCCATGTTG ATCTTCACTC TTCTCACCAC AAACTCCCTT CCTTTCTTCT CAAACAGCCT TTTGGGCTCG

*Ricinus communis* ATTCGAAATT GTTCATGTTG ATCTTGATTC TGGAGAGCAT AAGCGATCTG AATTCCTTCT CAAACAGCCA TTTGGGCAAG

*Jatropha curcas* TTTTCAAATT ATAACTCTTG ATCTCAAATC CGGGGACCAT AAGCATCCTG ACTTCCTTCT CAAACAGCCG TTTGGGCAAG

*Hevea brasiliensis* GTTTGAGATT CTTCATGTTG ATCTTGCTTC TGGAGAGCAC AAACGACCTG ACTTCCTTCT CAAACAGCCA TTTGGGCAAG

*Manihot esculenta* ATTTGAGATT GTTCAGCTTG ATCTTGATTC CGGCGAGCAA AAGCAACCTC ACTTCCTTCT CAAACAGCCA TTTGGCCAAG

*VvGST4* GTTTGAGGTT GTCCATGTCG ACCTCGACTC TGGCGAGCAA AAACGGCCTG ATTTCCTCCT TCGACAGCCT TTTGGGCAAG

*PpRiant1* TTTCGAAATT GTTGATGTTA ATCTTGAGGT GGGAGAGCAA AAGCAACCTC AGTTCCTCTC CCGTCAGCCG TTTGGTCAAG

*PpRiant2* TTTCGAAATT GTTGATGTTA ATCTTGAGGT GGGAGAGCAA AAGCAACCTC AGTTCCTCTC CCGTCAGCCG TTTGGTCAAG

*CkmGST3* CTTTGAAATC ATTCACGTCG ACCTTGAATC CGGGGAGCAT AAGAAGCCCG ATTTTCTTCT TCGCCAGCCC TTTGGACAAG

*PhAN9* TTTTGAGCTT ATCCATGTTG ATCTTGATTC TCTCGAGCAG AAAAAACCTG AGTTTCTAGT TTTACAGCCA TTTGGACAAG

*AtTT19* ATTTGAGATT ATTCATATCG ATCTTGATAC ATTTGAGCAA AAAAAACCAG AACATCTTCT TCGTCAGCCA TTTGGTCAAG

*LcGST4* ATTCGAAATC ATTCATGTTA ATATCGACAA TGGAGAGCAT AAGCGTCCTG AGTTTCTTCT TCGACAGCCT TTTGGGCAAG

....|....| ....|....| ....|....| ....|....| ....|....| ....|....| ....|....| ....|....|

165 175 185 195 205 215 225 235

*Bract1* TTCCAGCTAT AGAAGATGGG GATTTCAGGC TTTTTGAATC AAGAGCCATA ATGAGATACT ATGCAACAAA ATATGAAGAA

*Euphorbia esula* TTCCAGTCAT AGAAGATGGA GATTTAAGGC TTTTTGAATC AAGGGCCATA ATGAGATACT ATGCAACAAA ATATGAAGAA

*Euphorbia pekinensis* TTCCGGTGGT TGAAGACGGA GATTTGAGGC TTTTTGAATC AAGGGCCATA ATGAGATACT ATGCAACAAA ATATGAAGAA

*Ricinus communis* TCCCGGTGGT AGAGGATGGA AATTTTCAGC TTTTTGAATC GAGAGCCATA GCAAGGTACT ACGCAACGAA ATATGCAGAT

*Jatropha curcas* TTCCGGTGGT AGAAGATGGC GATTTTCGAC TTTTTGAATC GAGGGCAATT TTGAGGTACT ATGCAGCCAA ATATGCAGAC

*Hevea brasiliensis* TTCCTGTCGT GGAGGAGGGT GATTTAGAGC TTTTTGAATC TAGGGCAATC ATAAGGTACT ACGCAGCGAA ATACGCAGAC

*Manihot esculenta* TTCCAGTCGT AGAGGAGGGT GATTTCCAGC TTTTTGAATC TAGGGCAATC ATAAGGTACT ACGCATCGAA GTATGCTGAT

*VvGST4* TTCCAGTGGT AGAAGATGGC GATTTCAGGC TGTTTGAGTC GAGGGCGATT GTGAGGTACA TCGCGGCCAA GTACGCGGAG

*PpRiant1* TTCCAGCAGT AGAAGATGGT GATTTCAGGC TATTTGAGTC TAGGGCTATT ATCAGATACT ACGCAGCCAA GTACGCCGAC

*PpRiant2* TTCCAGCAGT AGAAGATGGT GATTTCAGGC TATTTGAGTC TAGGGCTATT ATCAGATACT ACGCAGCCAA GTACGCCGAC

*CkmGST3* TCCCAGCCAT AGAGGATGGA GATTTCAGGC TTTTCGAGTC CCGGGCAATC ATGAGATACT ATGCAGCAAA GTATTCCGAG

*PhAN9* TTCCTGTCAT TGAAGATGGT GATTTTAGGC TTTTTGAATC CAGAGCAATA ATTAGGTACT ATGCAGCAAA GTATGAAGTC

*AtTT19* TTCCAGCCAT AGAAGATGGA GATTTCAAGC TTTTTGAATC ACGAGCCATC GCGAGATACT ACGCTACCAA GTTCGCGGAC

*LcGST4* TTCCAGTAAT TGAAGATGAC GATTTTAAGC TTTTTGAATC AAGGGCAATA CTGCGGTACT ACGCGGCCAA GTATGCGGAC

....|....| ....|....| ....|....| ....|....| ....|....| ....|....| ....|....| ....|....|

245 255 265 275 285 295 305 315

*Bract1* AGAGGGCCCA ATTTGTTAGG AAAAACATTA GAAGAGAAAG CAATAGTTGA TCAATGGGTT GAAGTGGAAG CCCATAATTT

*Euphorbia esula* AGGGGCCCAG ATTTGGTAGG GAAAACATTA GAAGAAAAAG CAATAGTGGA TCAATGGGTT GAAGTGGAGG CCCATAACTT

*Euphorbia pekinensis* AGAGGGCCCA ATTTGATAGG GAAAACATTA GAAGAGAAAG CAATAGTGGA TCAATGGGTT GAAGTGGAGG CCCATAACTT

*Ricinus communis* CGTGGGGCAA ACCTTCTAGG AACAACACTG GAAGAGAGAG CAATAGTGGA CCAATGGCTC GAAGTAGAAG CACACAACTT

*Jatropha curcas* AGAGGGCCAA ACCTACTGGG CAAAACCCTA GAAGATAAAG CTTTAGTAGA TCAATGGCTT GAAGTCGAAG CACACAACTT

*Hevea brasiliensis* CGTGGGCCTA ACCTAGTTGG AAAGACATTG GAAGAGAGAG CTCTAGTGGA TCAATGGCTG GAAGTGGAAG CCCACAACCT

*Manihot esculenta* CGTGGATCAA ATCTACTTGG TAATACATTC GAAGAGAGAG CTGTGGTGGA TCAATGGCTA GAAGTGGAAG CCCACAACTT

*VvGST4* CAAGGGCCTG ACCTCTTGGG AAAAAGCTTG GAGGAGAAAG CGGTAGTTGA TCAATGGCTG GAAGTGGAAG CTCACAACTT

*PpRiant1* CGTGGTCCCA ACCTATTGGG AACAACCCTG GAGGAGAAGG CTCTGGTGGA TCAGTGGCTG GAAGTAGAAG CTCACAATTT

*PpRiant2* CGTGGTCCCA ACCTATTGGG AACAACCCTG GAGGAGAAGG CTCTGGTGGA TCAGTGGCTG GAAGTAGAAG CTCACAATTT

*CkmGST3* AAGAATCCTG ACCTACAGGG ATCGACTCTA GAGGAGAAAG CTCTAGTTGA CCAGTGGCTC GAGGTCGAAT CACACAACTT

*PhAN9* AAGGGAAGCA AACTAACAGG AACAACATTG GAAGAGAAAG CTCTAGTTGA TCAATGGCTA GAAGTTGAAT CCAATAACTA

*AtTT19* CAAGGCACGA ACCTTTTGGG CAAGTCTCTA GAGCACCGAG CCATCGTGGA CCAGTGGGCT GACGTGGAGA CCTATTACTT

*LcGST4* CGTGGACCCA ACCTGCTTGG AACCACCCTG GAGGAGAGGG CCAGGGTGGA CCAGTGGCTG GAAGTGGAAG CACACAACTT

....|....| ....|....| ....|....| ....|....| ....|....| ....|....| ....|....| ....|....|

325 335 345 355 365 375 385 395

*Bract1* CAATAATTTG GTTTACAATA TTGTAATTGA AGTTTTGATA AAGCCA---A AAATGGGGGA ACAAGGTGAC ATCAACATAG

*Euphorbia esula* CAATGAATTG GTATACACTA TTGTGATTCA AGTTTTGATA AGGCCC---A AAATGGGGGA ACGAGGGGAC ATGAGTTTAG

*Euphorbia pekinensis* CAATGGATTG GTTTACAACA TTGTGGTTCA AGTTTTGATA AGGCCC---A AGATGGGTGA AGAAGGAGAC ATGAAATTAG

*Ricinus communis* CAACGAATTG GTGTACAACC TGGTGCTTCA ACTAGTGATC TTTCCG---C GAATGGGACA GCCAGGGGAC CTAAAATTAG

*Jatropha curcas* CAACGACCTG GTCTACAATA TTGTGCTTCA ACTTGTCGTC TTTCCT---C AGATGGGGCA AGCTGGAGAC CCAAAGGTAG

*Hevea brasiliensis* CAACGATTTG GTGTACAATC TGGTGTTTCA ACTTGTGATC TTGCCA---A GGATGGGGCA GCCAGGGGAC TTGAAGTTAG

*Manihot esculenta* CAATGATTTG GTGTACAATC TGGTGTTTCA ACTCGTGATT CTGCCG---A GAATGGGGCA GCACGGGGAC TTGAAGTTAG

*VvGST4* CAACGAGTTG GTGTACACAC TGGTCATGCA GCTAGTGATC CTACCT---C GAATGGGTGA GCGGGGGGAC TTGGCTTTAG

*PpRiant1* CAATGACTTG GTTTACACTC TGGTACTTCA ACTTCTGGTG CTGCCT---C GCATGGGGGA GCGTGGTGAC GTGGCCTTGG

*PpRiant2* CAATGACTTG GTTTACACTC TGGTACTTCA ACTTCTGGTG CTGCCTGATC GCATGGGGGA GCGTGGTGAC GTGGCCTTGG

*CkmGST3* CAATGACCTG GTATACACTC TAGTACTCCA CCTCATGGTT TTCCCT---C AGATGGGCAA GCGCAGTGAC ATGCAGTTGG

*PhAN9* CAATGACTTA GTATACAACA TGGTCCTCCA ACTCCTAGTA TTCCCC---A AAATGGGACA AACCAGTGAC TTAACATTGG

*AtTT19* CAACGTTCTG GCCCAACCCC TCGTGATTAA CCTAATCATC AAGCCT---A GGTTAGGCGA GAAATGTGAC GTCGTTTTGG

*LcGST4* CAATGACTTG ATCTACACTA TGGTGCTTCA ACTGATAGTT ATTCCA---A GCATGGGGCA GCCTGGGGAC CTGACGTTGG

....|....| ....|....| ....|....| ....|....| ....|....| ....|....| ....|....| ....|....|

405 415 425 435 445 455 465 475

*Bract1* TCAAAAGCTG TGAACATAAG CTGGATAAAG TGTTCGATGT GTACGAGGAA AGGCTATCCA GTTCCAAATA TCTTGGAGGA

*Euphorbia esula* TCAAAACCTG CGAGCATAAG CTGGGGAAAG TGTTGGATGT GTACGAGGAA AGGTTATCTA GTTCCAAGTA TCTTGGAGGA

*Euphorbia pekinensis* TCAAAAGCTG CGAGCGAAAG CTAGAGAAAG TGTTCGATGT GTACGAGGAA AGGTTGTCCC ATTCCAAGTA TCTTGGAGGA

*Ricinus communis* TCCACAACTG CGAGCAAAAA TTAGAGAAGG TGTTCGACAT CTACGAGAAG AGATTGTCCA AGACCAAATA CCTTGCTGGA

*Jatropha curcas* TGCAGAACTG CGAGCAAAAG CTGGAGGAAG TATTAGACAT ATACGAGCAA AGATTGGCGA CGAGCAAATA TCTTGCCGGA

*Hevea brasiliensis* TCCACAGCTG CGAGCAGAAG CTGGAGCAGG TGCTCGATGT GTATGAGCAA AGATTGTCCA AGAGCAAGTA TCTTGCTGGA

*Manihot esculenta* CCCGCAGCTG TGAGCAGAAG CTAGAGAAGG TGCTAGATGT GTATGAGCAA AGGTTGTCCA AAAGCAAGTA TCTTGCTGGA

*VvGST4* CCCACACTTG TGAGCAGAAG CTGGAAAAGG TGTTTGATGT GTATGAGCAG AGGCTGTCGA AGAGCCGGTA CCTTGCAGGA

*PpRiant1* TCCATGCATG TGAGGAGAAA CTGGAGAAGG TGTTCGATGT TTATGAGGAA AGATTATCAA AGAGCAGCTA TCTGGCTGGA

*PpRiant2* TCCATGCATG TGAGGAGAAA CTGGAGAAGG TGTTCGATGT TTATGAGGAA AGATTATCAA AGAGCAGCTA TCTGGCTGGA

*CkmGST3* TACAAGAATG CGAGAGCAAA CTTGAGAAAG TATTTGATAT ATACGAGGAG AGATTGTCGA AGAGTAACTA CCTGGCCGGA

*PhAN9* TAACAAAATG TGCCAACAAG TTAGAGAATG TCTTTGACAT TTATGAACAA AGGTTGTCAA AGAGTAAATA TCTAGCAGGA

*AtTT19* TCGAGGATCT CAAAGTGAAG CTAGGAGTGG TCTTGGACAT ATACAATAAC CGGCTTTCTT CGAACCGGTT TTTGGCTGGT

*LcGST4* TCCACTCCTG CGAGCAGAAG CTAGAGGCAG TTTTTGACGT CTACGAAAAG CAGCTTTCGA AGAGTAAGTA CCTCGCCGGA

....|....| ....|....| ....|....| ....|....| ....|....| ....|....| ....|....| ....|....|

485 495 505 515 525 535 545 555

*Bract1* GATTATTTCA CACTTGCTGA TTTAACCCAT ATGCCTTCCA TTAGGTACCT TGTTCATGAG CTTGGGTTAG CCCATTTGGT

*Euphorbia esula* GATCATTTCA CACTTGCTGA TTTAACCCAT ATGCCTTCTC TTAGATACCT TGTTAATGAA CTTGGGTTAA CCCATTTGGT

*Euphorbia pekinensis* GATCATTTCA CAATTGCTGA TTTAACACAT ATGCCTTCTA TGAGATATCT TGTAGATGAG CTTGGGTTAA GCCATTTGGT

*Ricinus communis* GATTACTTCA CACTTGCTGA TTTAAGCCAT TTGCCTGCCA TTAGATACCT TGTCAATGAT GCTGGATTAG GGCATTTGGT

*Jatropha curcas* GATTATTTCA GTCTTGCTGA TTTAAGCCAT ATGCCTGCAA TTAGATACCT TGTGGATGAA GTTGGGAAAG GGCATTTGGT

*Hevea brasiliensis* GATTCATTTA CTCTTGCTGA TTTGAGCCAT TTGCCTGGAA TTAGATATCT GGTGAATGAA GTTGGAATGT GCCATTTGGT

*Manihot esculenta* GAATCATTTA CACTTGCTGA TTTGAGCCAT TTGCCTGGCA TTAGATACCT GGTGAATGAA GCTGGAATGT ATCATTTGGT

*VvGST4* GATTCATTCA CTCTCGCTGA TCTGAGTCAT CTTCCGGCCA TCAGATACTT GGTGAAGGAA GCTGGAATGG CGCACTTGGT

*PpRiant1* GAAGCTTTCA CTCTGGCTGA TCTGAGCCAT CTTCCAGGGA TAAGCTATCT GATTGATGAA GCTAAATTGG GGCATTTGGT

*PpRiant2* GAAGCTTTCA CTCTGGCTGA TCTGAGCCAT CTTCCAGGGA TAAGCTATCT GATTGATGAA GCTAAATTGG GGCATTTGGT

*CkmGST3* AAATTGTTCA CCCTTGCCGA CCTCAGCCAC CTCCCATCTA TCACTTTTCT AATGGGCGAG GGTGGGTTGG GACATATGGT

*PhAN9* GAGTTTTTCT CACTAGCTGA TCTAAGTCAC CTTCCTAGTT TAAGGTTCTT AATGAATGAA GGTGGTTTTT CACATTTGGT

*AtTT19* GAAGAATTCA CTATGGCTGA TTTGACGCAC ATGCCGGCGA TGGGGTACTT GATGAGTATA ACCGATATAA ACCAGATGGT

*LcGST4* GATTGGTTCT CTTTGGCAGA CCTCAGCCAC ATGCCTGCCC TCCGGTTCTT GATGGAGGAT GCTAAGTTGG TGCACCTGGT

....|....| ....|....| ....|....| ....|....| ....|....| ....|....| ....|....| ....|....|

565 575 585 595 605 615 625 635

*Bract1* TCACAATAGA AACAAGGTCA ATGCTTGGTG GATTGATATA TCGGACCGAC CGGCTTGGAA AAATTTGATG ATTCTTGCTG

*Euphorbia esula* TCAAAATAGG AAGAATGTCA ATGCTTGGTG GACTCATATT TCTAGCCGTC CTGCTTGGAA AAAATTGATG ATTCTTGCTG

*Euphorbia pekinensis* TCAAAATAGG AAGAATGTGA ATGCTTGGTG GAATAATATT TCTAACCGTC CTGCTTGGAG AAAATTGATG ACTCTCGCTT

*Ricinus communis* GACAGATAGG GAGAAAGTGA ACGCCTGGTG GGAGGATATT TCTAGCCGTC CGGCTTGGAA AAAGTTAATG AAACTTGCTG

*Jatropha curcas* GAGAGAGAGG AAGAAGGTTA ATGGCTGGTG GGAAGATATC TCAAGCCGTC CTGCTTGGAA GAAATTAATG GAGCTTTGTG

*Hevea brasiliensis* GAGAGAGAGG GAGAATGTTA ATGCTTGGTG GCAGGATATT TCAAGCCGTC CAGCTTGGAA GAAATTAATG GAACTTGCTG

*Manihot esculenta* GAAAGAGAGG GAGCATGTTA ATGCTTGGTG GCAGGATATT TCAAGCCGTC CTGCATGGAA GAAATTGATG GAACTTGCTG

*VvGST4* TACTGAGAGG AAGAGTGTGA GTGCATGGTG GGAGGACATT TCCAACAGGG CTGCTTGGAA AAAAGTCATG GAGCTCGCTG

*PpRiant1* GTCTGAGAGG AAGAATGTGA ATGCTTGGTG GAAAGACATA TCCAACAGGC CTGCTTGGAA GAAACTAATG AGCCTTGCTA

*PpRiant2* GTCTGAGAGG AAGAATGTGA ATGCTTGGTG GAAAGACATA TCCAACAGGC CTGCTTGGAA GAAACTAATG AGCCTTGCTA

*CkmGST3* GAGGAACAGA AAGAACGTCA ACTCGTGGTG GATGGATATT TCGAGCAGGC CTTCTTGGAA GAAGGTGCGG AAGCTGATGG

*PhAN9* GACCAAGAGA AAGTGTTTGC ATGAGTGGTA TTTGGATATT TCAAGTAGGG ATTCTTGGAA GAAAGTGTTG GACCTCATGA

*AtTT19* TAAGGCTCGG GGTAGTTTTA ACCGGTGGTG GGAAGAGATT TCGGATAGAC CGTCTTGGAA GAAGCTTATG GTGCTGGCTG

*LcGST4* GAAGGAGAGG AAGCATGTTA ATGCGTGGTG GGAGGAGATT TCCGGCCGCC TCTCGTGGAA GAAATTGATG AAGCTTGCCT

....|....| ....|....| ....|....| ....|....| ....|....| ....|....| ..

645 655 665 675 685 695

*Bract1* G---TTATTA G--------- ---------- ---------- ---------- ---------- --

*Euphorbia esula* GATTTGATTG A--------- ---------- ---------- ---------- ---------- --

*Euphorbia pekinensis* CCACCCATTA G--------- ---------- ---------- ---------- ---------- --

*Ricinus communis* G---TTATTA G--------- ---------- ---------- ---------- ---------- --

*Jatropha curcas* G---TTACTG A--------- ---------- ---------- ---------- ---------- --

*Hevea brasiliensis* G---TTTCTA G--------- ---------- ---------- ---------- ---------- --

*Manihot esculenta* G---TTTCTA G--------- ---------- ---------- ---------- ---------- --

*VvGST4* CTTGA----- ---------- ---------- ---------- ---------- ---------- --

*PpRiant1* GTGACTACTA G--------- ---------- ---------- ---------- ---------- --

*PpRiant2* GTGACTACTA G--------- ---------- ---------- ---------- ---------- --

*CkmGST3* ACTAG----- ---------- ---------- ---------- ---------- ---------- --

*PhAN9* TGAAGAAGAT ATCAGAGATT GAAGCAGTGT CTATCCCAGC TAAAGAAGAA GCAAAAGTTT GA

*AtTT19* GTCACTGA-- ---------- ---------- ---------- ---------- ---------- --

*LcGST4* ACTATTAG-- ---------- ---------- ---------- ---------- ---------- --
